# Asymmetric Unimodal Maps with Non-universal Period-Doubling Scaling Laws

**DOI:** 10.1007/s00220-020-03835-9

**Published:** 2020-08-18

**Authors:** Oleg Kozlovski, Sebastian van Strien

**Affiliations:** Imperial College, London, UK

## Abstract

We consider a family of strongly-asymmetric unimodal maps $\{f_t\}_{t\in [0,1]}$ of the form $f_t=t\cdot f$ where $f:[0,1]\rightarrow [0,1]$ is unimodal, $f(0)=f(1)=0$, $f(c)=1$ is of the form and

$$\begin{aligned} f(x)=\left\{ \begin{array}{ll} 1-K_-|x-c|+o(|x-c|)&{} \text{ for } x<c, \\ 1-K_+|x-c|^\beta + o(|x-c|^\beta ) &{} \text{ for } x>c, \end{array}\right. \end{aligned}$$

where we assume that $\beta >1$. We show that such a family contains a Feigenbaum–Coullet–Tresser $2^\infty$ map, and develop a renormalization theory for these maps. The scalings of the renormalization intervals of the $2^\infty$ map turn out to be super-exponential and non-universal (i.e. to depend on the map) and the scaling-law is different for odd and even steps of the renormalization. The conjugacy between the attracting Cantor sets of two such maps is smooth if and only if some invariant is satisfied. We also show that the Feigenbaum–Coullet–Tresser map does not have wandering intervals, but surprisingly we were only able to prove this using our rather detailed scaling results.

## Introduction

The theory of one-dimensional dynamics is rather well developed. Especially a lot is known for smooth one-dimensional unimodal maps (*i.e. *maps of an interval having just one critical point): absence of wandering intervals, real bounds, convergence of renormalizations, density of hyperbolic maps, various scaling properties, etc... Most of these results are obtained under some conditions on the order of the critical point, typically the map is assumed to be smooth or even analytic, and the critical point is assumed to be non-flat and in many results the order is, additionally, assumed to be an even integer. Moreover, in these studies, the order of the critical point is assumed to be the same on both sides, *i.e. *in a small neighbourhood of the critical point the map behaves as $f(x)-f(c) \sim -K|x-c|^\alpha$, where *c* denotes the critical point and $\alpha$ is its order. Here $\sim$ means that the left hand side divided by the right hand side tends to 1 as $x\rightarrow c$.

A natural generalisation and the next step in the theory of one-dimensional maps is to consider maps which have different critical orders on the two sides of the critical point. Specifically, to study maps such that near the critical point the map *f* takes the form

$$\begin{aligned} f(x)-f(c) \sim \left\{ \begin{array}{rl} - K_-|x-c|^\alpha &{} \text{ for } x<c\\ - K_+|x-c|^\beta &{} \text{ for } x>c\end{array} \right. \end{aligned}$$

where $1\le \alpha \le \beta$. Maps for which $\alpha <\beta$ deserve to be studied on their own merit and can appear in applications, *e.g. *the Poincare first return maps of smooth two-dimensional flows or semi-flows can have singularities with different critical order. We will call these maps *strongly asymmetric* when $\alpha <\beta$ and *weakly symmetric* when $\alpha =\beta$.

The purpose of this project is to ask the following question: do strongly asymmetric maps have substantially different properties when compared with ‘symmetric’ unimodal maps? In some cases the answer would be *no*. For example, hyperbolic maps will have similar properties because the order of the critical point does not play any role for such maps. A slightly less trivial example is the case of Misiurewicz maps (that is maps whose critical orbit does not accumulate on the critical point) where the standard theory of one-dimensional maps can be applied to strongly asymmetric maps without any significant alteration.

At the start of this project on strongly asymmetric maps, the authors were not sure what to expect in non-trivial cases. For example, could one expect universality? Could there be wandering intervals?

In this paper we will make a first step towards a general theory for such maps by considering one of the simplest non-trivial class of such maps, namely infinitely renormalizable maps of the Feigenbaum–Coullet–Tresser combinatorics and will show that the scaling properties and limits of renormalizations are quite different compared to the classical ones. Note that the theory of such infinitely renormalizable maps is still far from complete even in the case of maps with a ‘symmetric’ critical point when the order of the critical point is not an even integer. Though it is generally believed that the renormalizations should converge in this case, no proof is known.

Before we formulate our results, let us quickly discuss some obvious differences between symmetric and asymmetric cases in the setting of the Feigenbaum–Coullet–Tresser maps (which we will often call $2^\infty$*maps* or *maps of* $2^\infty$*combinatorics*). Recall that for such a map one can construct a shrinking sequence of intervals $[a_n, b_n]$ around the critical point such that the restrictions $f^{2^n}|_{[a_n,b_n]}$ are unimodal maps (also with $2^\infty$ combinatorics) for $n=0,1,\dots$. Let $R_n: [a_n, b_n] \rightarrow [0,1]$ be linear surjections and let the *n*-th renormalizations ${{\tilde{f}}}_n$ of *f* be defined by the formula ${{\tilde{f}}}_n= R \circ f^{2^n} \circ R^{-1}$.

When the order of the critical point of *f* is an even integer it is known that the sequence of the renormalizations converges to some unimodal real-analytic map which is universal in the sense that this limit map depends only on the order of the critical point and not on the particular choice of the initial map *f*, for references see below.

Now let us check what happens with renormalizations when the map is strongly asymmetric. First, note that the renormalization intervals $[a_n,b_n]$ can be constructed in different ways. These differences are non essential, and we will find if convenient to assume that $f(a_n)=f(b_n)$. Then asymptotically we have $|a_n-c| \sim (K_+/K_-)^{\frac{1}{\alpha }} |b_n-c|^{\frac{\beta }{\alpha }}$, and since $\alpha <\beta$ we see that $|a_n-c| \ll |b_n-c|$. Thus, the critical point is located much closer to the left end of the renormalization intervals and in the limit after rescaling the critical point coincides with the left boundary point of the rescaled interval. This means that the renormalizations cannot converge to a unimodal map! As we will see in the case we consider, when $\alpha =1<\beta$, the limit of ${{\tilde{f}}}_n$ exists (even though it is degenerate), and moreover is universal in the sense that it only depends on $\beta$. There is even an explicit formula for it!

To initiate this research direction we decided to focus on strongly asymmetric unimodal maps with Feigenbaum–Coullet–Tresser combinatorics. Though the authors believe that these results must hold in the general case $1\le \alpha < \beta$, we were only able to prove them under the assumption that $\alpha =1$ because in that case we are able to use the notion of *semi-extension* which is defined in Sect. 8.1 and discussed a little more in the informal summary below. The precise definition of the class of considered maps is given in Sect. 2.

### Informal summary of the the results in this paper

**Fig. 1:**
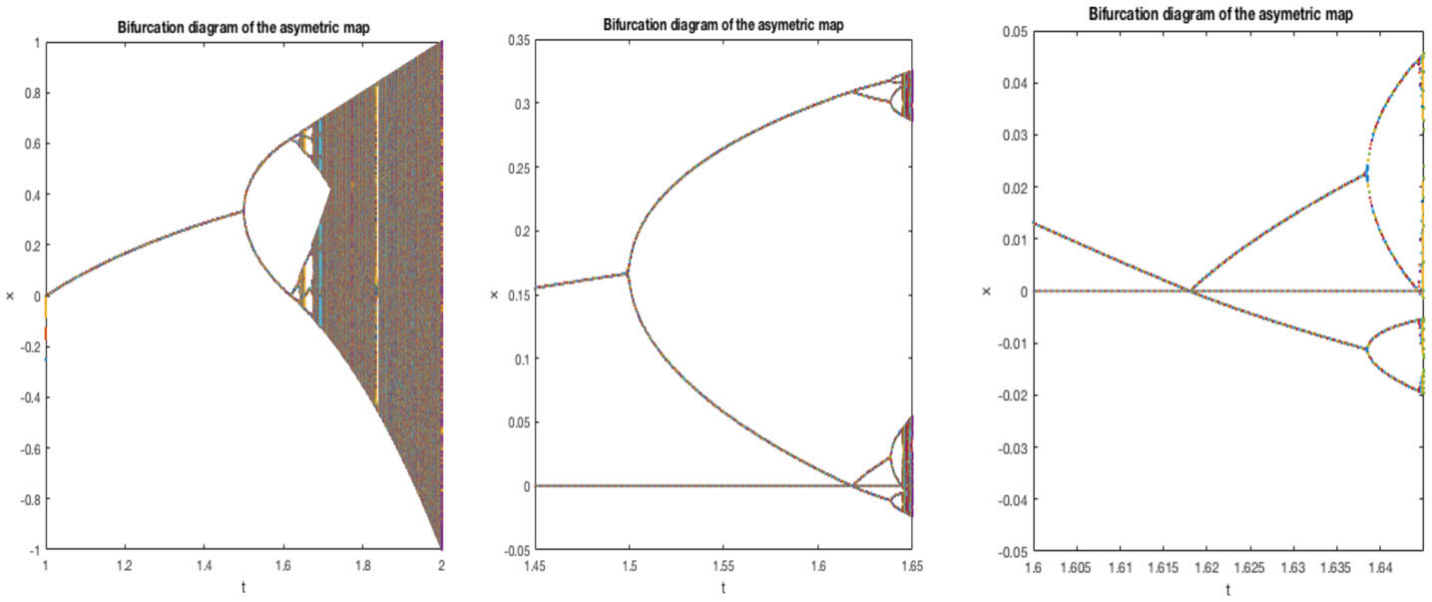
The bifurcation diagram of the family of asymmetric maps $\{f_t\}_{t\in [1,2]}$, defined in (4) together with two zoomed-in versions with the position of the critical point $x=0$ marked. Note that the doubling bifurcation from period $2^n$ to period $2^{n+1}$ when *n* is odd is not the classical one; in the current asymmetric case the period doubles precisely when 0 is periodic (rather than when the multiplier is equal to $-1$), as is explained in Theorem 11. The parameter scalings also appears to be rather different than that for the quadratic family

- We study bifurcations leading to a Feigenbaum–Coullet–Tresser map and prove the existence of such a map in our class (Theorem 1). The argument here will be rather soft. Although the period doubling diagram, see Fig. 1, looks qualitatively the same as for the quadratic family, there are important differences when $1=\alpha <\beta$: when *n* is odd, the periodic orbit of period $2^n$ doubles its period when it contains the critical point rather than when its multiplier is $-1$.
- An initial crucial step in the theory of unimodal (or, more generally, one-dimensional) maps is to establish the existence of distortion bounds. This usually relies on ‘real bounds’ or ‘Koebe space’, by which we will mean, in this setting, that the first entry map $f^{2^n-1}$ from the critical value to $[a_n,b_n]$ has a diffeomorphic extension whose range contains a definite intervals around $[a_n,b_n]$. Having this property gives distortion bounds on the first entry map. Surprisingly, as we will show such extensions do NOT exist for $f^{2^n-1}$ (Theorem 2, also Theorem 9). As far as we know this is the first type of unimodal map for which such bounds are known not to exist. In spite of the absence of Koebe space, we will be able to control the distortion of certain branches of the iterates of *f* (Theorem 3). This is the main step in this paper, and the proof is involved and interesting. For an idea of the proof see Sect. 8.3. Here we rely heavily on the fact that *f* is almost linear on one side of the critical point. This lead us to invent the notion of a *semi-extension*, see Sect. 8.1. This means that we consider a maximal diffeomorphic extension of $f^n$ which is obtained by taking an appropriate composition of the right branch of *f* and a (diffeomorphic) extension of the left branch of *f* beyond the critical point. Using this tool, we analyse various scenarios concerning the position of certain points which as such have no dynamical interpretation. Thus we obtain increasingly precise information, and thus we eventually obtain extremely good real bounds for these semi-extensions.
- Using distortion properties mentioned above, we are able to obtain very precise scaling laws, see Theorem 4. These scaling laws are rather different than for the usual ‘symmetric’ Feigenbaum–Coullet–Tresser case where the scalings are geometric and universal (the rates only depend on the order of the critical point) and so we have
  $$\begin{aligned} |b_{k+1}-a_{k+1}| \sim \kappa |b_k- a_k| \end{aligned}$$
  for some $0<\kappa <1$ which does not depend on which unimodal map one takes (provided its critical point is quadratic). In our setting, the scalings of their lengths are quite different for even and odd steps, namely
  $$\begin{aligned} |b_{2k+2}-a_{2k+2}|\sim & {} \beta ^{\frac{-2}{\beta -1}} K_0^{\frac{1}{\beta -1}} \lambda ^{-2} | b_{2k+1}-a_{2k+1}|^2 \\ |b_{2k+1}-a_{2k+1}|\sim & {} \lambda |b_{2k}-a_{2k}| \end{aligned}$$
  where $\lambda$ is the root of
  $$\begin{aligned} \lambda ^\beta +\lambda -1=0 \end{aligned}$$
  and $K_0=K_+/K_-$. Moreover, there exists $\Theta >0$ so that
  1 $$\begin{aligned} |b_{2k}-a_{2k}| \sim \beta ^{\frac{2}{\beta -1}} K_0^{\frac{-1}{\beta -1}} \exp (-2^k \Theta ). \end{aligned}$$
- In the classical Feigenbaum–Coullet–Tresser $2^\infty$ case, maps with quadratic critical points are necessarily differentiably conjugate along the closure of the forward iterates of the critical point. This phenomenon is usually referred to as *universality*. Here this universality no longer holds: two maps $f,{{\tilde{f}}}$ are Lipschitz (and even differentiably conjugate) if and only if
  $$\begin{aligned} \beta ={{\tilde{\beta }}} , \Theta = {{\tilde{\Theta }}}. \end{aligned}$$
  This means that this case is rather more similar to [37, 42] where there are also necessary and sufficient conditions for these maps to be differentiably conjugate at the turning point, see Theorems 1 and 7. One of the consequences of this fact is that *f* and its renormalizations are not Lipschitz conjugate even at the critical point *c*.
- In the ‘symmetric’ case the *n*-th renormalization of the function converges to some analytic function with unknown closed formula. Here we obtain a degenerate limit, but whose form is entirely explicit, see Theorems 5 and 6.
- The $2^\infty$ maps we consider do not have wandering intervals, see Theorem 10. Absence of wandering interval for our class of maps implies that the maps we consider are all topologically conjugate to the quadratic Feigenbaum–Coullet–Tresser map.

### History of the problem

Renormalisation and rigidity results were proved previously for circle diffeomorphisms with Diophantine conditions on the rotation number [19, 67]. For circle maps with discontinuities of the derivative (break type singularities) there are quite a few results, see e.g. [1, 9, 21–24]. For smooth homeomorphism of the circle with a critical point, there are results by [2, 12, 13, 25, 65]. For infinitely renormalizable unimodal interval maps there is a rich history, starting with the conjectures of Feigenbaum and Coullet-Tresser. Rigorous proofs were finally provided by [3, 48, 49, 60], see also [14, 30, 56–58]. The weakly symmetric case is considered in [52]. Note that for interval maps smooth rigidity is not possible, so the natural context there is quasi-symmetric rigidity. This was proved in increasing generality in [7, 17, 28, 35], see also [5, 7, 28, 33] and [8, 27, 29, 55], using the notion of polynomial-like mappings, see [11]. For Lorenz maps there is another very interesting phenomenon: in this case the renormalization operator can have several (degenerate) fixed points even when the left and right critical exponent at the discontinuity is the same. This can happen even for bounded combinatorics, and return maps can degenerate [44, 64] see also [43, 45].

For circle maps with plateaus see for example [16, 40, 42, 53, 54, 61–63]. Here it is also natural to explore the role of the orders of the critical points at the boundary points of a plateau [*a*, *b*]. Quite often it is assumed that these orders are the same, see [40, 53, 54, 61] but not in the entire literature, see for example [16, 62, 63]. For such maps, super-exponential scaling was obtained in [16] under the assumption that $f(x)-f(a)\sim -|x-a|$ to the left of *a* and $f(x)-f(b)\sim |x-b|^\beta$ with $\beta >1$ to the right of *b*. Here the $q_n$-th iterates of the plateau are considered, and these iterates converge super-exponentially in terms of *n*. In [53, 54] it is assumed that $f(x)-f(a)\sim -|x-a|^\alpha$ to the left of *a* and $f(x)-f(b)\sim |x-b|^\alpha$ to the right of *b* (so the orders on both sides are the same). The main result in [53] is that one has bounded geometry (so the approach rate is at most exponential) in terms of *n* if $\alpha >2$, and a super-exponential approach is if $\alpha \le 2$. In [54] it is shown that any two such maps with bounded geometry and with the same rotation number, are quasi-symmetrically conjugate.

The question whether two maps which are combinatorially the same, are in fact topologically conjugate hinges on absence of wandering intervals. The first results in this direction were obtained for circle diffeomorphisms in the 1920’s by Denjoy [10], for critical circle maps in [66] and for circle maps with plateaus in [40]. For interval maps there are results, in increasing generality, [4, 18, 34, 41, 46, 50, 59]. On the other hand, interval exchange transformations can have wandering intervals, see e.g. [38]. Furthermore, it is not known whether a circle homeomorphism with a strongly asymmetric critical point (which means that $f(x)-f(c)\sim -|x-c|^\alpha$ to the left of *c* and $f(x)-f(c)\sim |x-c|^\beta$ to the right of *c* where for example $1\le \alpha <\beta$) can have wandering intervals. It was for this reason that the authors were curious to find out whether one can have wandering intervals in the strongly asymmetric case.

#### Open questions.

Before stating our results rigorously, let us discuss questions and possible directions for further research.

**Super-exponential scaling when** $1<\alpha <\beta$. In this paper we always assumed that the left critical order $\alpha$ of our map is equal to 1. We believe that the super-exponential scaling of the points $a_n$ and $b_n$ that we have shown here, also holds when $1<\alpha <\beta$. Indeed, the strong asymmetry (and the fact that the map is unimodal) forces there to be scalings of entirely different orders of magnitude: the scaling on the left side of the critical point is a power of the scaling on the right side of the critical point. Assuming suitable ‘real bounds’ (and that the map has $2^\infty$ dynamics) this implies super-exponential scalings. However, it is very unlikely that such real bounds hold when $\alpha <\beta$, and this is one reason why our proof is delicate. But if what we suspect is true, then the case $\alpha =\beta$ is completely different from when $\alpha <\beta$. The same phenomena should also hold for many other combinatorics provided, amongst other things, the critical point is accumulated from both sides under certain first return maps.

**Absence of wild attractors when** $1<\alpha <\beta$. It is well-known that in the ‘symmetric’ case, the so-called Fibonacci map has a wild attractor provided the order of the critical point is large. Inspired by our belief that one has super-exponential scaling, we believe that such attractors do not exist when $1<\alpha <\beta$, even if these numbers are arbitrarily large.

**Absence of wandering intervals.** In this paper we only proved absence of wandering intervals for the $2^\infty$ combinatorics and when $1=\alpha \le \beta$. We believe one has absence of wandering intervals without these assumptions. In fact, we tried and failed to prove this result in the case that $1<\alpha <\beta$.

**Monotonicity of bifurcations.** Notice numerical simulations suggest that the bifurcations from the family $f_t$ from Eq. (4) are monotone: no periodic orbit seems to disappear when *t* increases. When instead we consider the family

2 $$\begin{aligned} f_t(x)={\left\{ \begin{array}{ll} t-1 -t|x|^\alpha &{} \text{ when } x<0, \\ t-1- t x^\beta &{} \text{ when } x\ge 0 \end{array}\right. } \end{aligned}$$

with $\alpha ,\beta >1$ large, then there are partial results towards monotonicity in [31] see also [32]. Monotonicity for this family is only known in full generality when $\alpha =\beta$ is an even integer. For references on the history of results on monotonicity, see [32].

**More precise rigidity results.** Consider continuous degree one circle maps, which are smooth local diffeomorphisms outside a single plateau and with $x^\beta$ behaviour at the boundary points of this plateau. In earlier papers [40] it was shown that such maps have no wandering intervals, and in [53] it was shown that one has super-exponential decay of scales when $\beta \in (1,2)$ when the rotation number is golden mean. In [42], it is shown that there exist invariants for Lipschitz, differentiable and $C^{1+\epsilon }$ conjugacy. For related results see [6]. A similar obstruction to differentiable conjugacy also appears in [37].

**Parameter scaling.** Consider the family $f_t$ defined in (4) and let $t_n$ be the parameter where the turning point 0 has period $2^n$ for $f_{t_n}$ and let $t_*$ be so that $f_{t_*}$ has $2^\infty$ dynamics. Computer experiments suggest that the parameters $t_n$ scale also super-exponentially. We are hopeful that we will be able to elaborate the methods in this paper to prove the following

##### Conjecture 1

(Non-universality of parameter bifurcations).

3 $$\begin{aligned} |t_{n+2}-t_*| \sim \kappa |t_{n}-t_*|^2 \end{aligned}$$

where $\kappa$ depends non-trivially on the two parameters $\beta ,\Theta$ associate to the family $f_t$ and so is not a universal parameter, where $\Theta$ is defined through Eq. (1).

So we conjecture that, in our setting, the parameter scaling is super-exponential and non-universal. This is in contrast to the universality results for generic smooth families of unimodal maps with a quadratic critical point (where the genericity assumption is that the family is assumed to be transversal to the stable manifold of the renormalization operator) where one has the parameter scaling

$$\begin{aligned} |t_{n+2}-t_*| \sim \lambda |t_{n}-t_*| \end{aligned}$$

where $\lambda$ is universal and so does not depend on the family.

**Renormalisation theory in the smooth setting.** The renormalization theory we develop here is done by obtaining large bounds. This is quite different from the renormalization theory obtained for real analytic unimodal maps, [3, 14, 36, 48, 49, 60], see also [15, 39, 57]. Most of these results build on complex bounds and quasi-symmetric rigidity. For the most general results on these see [8, 11]. It would be interesting to tie these approaches together.

## The Setting of This Paper

Consider the class ${\mathcal {A}}_{\alpha ,\beta }$ of continuous unimodal maps $f : [a_0,b_0] \rightarrow [a_0,b_0]$ where $a_0<0<b_0$ and with the following properties:

1. $f(a_0)=f(b_0)=a_0$ and outside the turning point $c:=0$ the map *f* is $C^3$ and has Schwarzian derivative $Sf\le 0$. The authors believe that the results in this paper also hold without the $Sf\le 0$ assumption.
2. $c=0$ is the unique extremal value of *f* and $f'(x)>0$ for $x<0$ and $f'(x)<0$ for $x>0$.
3. Near the critical point $c=0$ the map *f* behaves as $f (x) \sim - K_- |x|^\alpha + f (0)$ for $x<0$ and |*x*| small and $f (x)\sim - K_+ x^\beta + f (0)$ for small positive values of *x*. The constants should satisfy $K_->0, K_+ > 0$ and $\beta >\alpha \ge 1$.

Almost everywhere in the paper we shall assume that $\alpha =1$, in this case we will denote ${\mathcal {A}}_{1,\beta }$ just by ${\mathcal {A}}$. We say that $f\in \mathcal A_{\alpha ,\beta }(2^\infty )$ if in addition

4 The map *f* has $2^\infty$ combinatorics, i.e. *f* is an infinitely renormalizable *Feigenbaum–Coullet–Tresser* period doubling map. By definition this means that there exists a shrinking sequence of intervals $[a_k,b_k]\ni c$ so that the restriction of $f^{2^k}$ to $[a_k,b_k]$ is again unimodal, mapping $\{a_k,b_k\}$ into itself and so that the intervals $f^i[a_k,b_k]$, $i=0,\dots ,2^k-1$ have pairwise disjoint interiors.

**Fig. 2:**
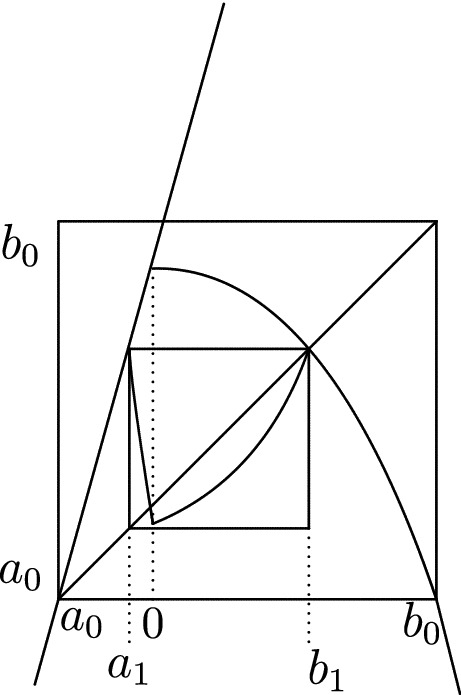
*f* together with its renormalization and its semi-extension

The sequence $[a_k , b_k ], k = 0, 1, . . .$, is constructed in the following way. Let $b_1$ be a fixed point of *f* with negative multiplier and $a_1$ be its preimage. Then $c_2 := f^2 (0) \in [a_1 , b_1 ]$. Notice that $a_0< a_1< 0< b_1 < b_0$. The intervals $[a_0,b_0]$ and $[a_1,b_1]$ are drawn in Fig. 2. Since the map *f* is assumed to be of Feigenbaum–Coullet–Tresser $2^\infty$ type, $f^2 |[a_1 ,b_1 ]$ is again unimodal; it decreases on $[a_1 , 0]$ and increases on $[0, b_1 ]$. The branch $f^2 |[a_1 ,0]$ has a fixed point which we will denote by $a_2$ and $b_2$ will denote its preimage by $f^2 |[0,b_1]$. Using again that *f* is a $2^\infty$ map, $f^4 |[a_2 ,b_2 ]$ is unimodal, and we can continue this process indefinitely and obtain a sequence of points $a_k<0<b_k$ and unimodal maps $f^{2^k}:[a_k,b_k]\rightarrow {[}a_k,b_k]$.

As will be shown in Theorem 11 in Sect. 6, there exist many maps within the class ${\mathcal {A}}(2^\infty )$. For example, there exists $t_*\in (1,2)$ so that $f_{t_*}\in {\mathcal {A}}(2^\infty )$ where $f_t:{[}-1,1]\rightarrow [-1,1]$, $t\in [1,2]$ is defined by

4 $$\begin{aligned} f_t(x)={\left\{ \begin{array}{ll} t(1+x)\,\, -1 &{} \text{ when } x<0, \\ t(1-x^\beta )-1 &{} \text{ when } x\ge 0. \end{array}\right. } \end{aligned}$$

As we will see in Sect. 6 this family $f_t$ undergoes unusual period doubling bifurcations, see Fig. 1.

**Some notation.** We say that the interval *T* is a $\tau$-scaled neighbourhood of $J \subset T$ if both components of $T\setminus J$ have at least size $\tau \cdot |J|$. We shall also use the notations

$$\begin{aligned} u_k\sim v_k&\iff \frac{u_k}{v_k}\rightarrow 1 \text{ as } k\rightarrow \infty \\ u_k\approx v_k&\iff 0<\liminf \frac{u_k}{v_k}\le \limsup \frac{u_k}{v_k} < \infty \text{ as } k\rightarrow \infty . \end{aligned}$$

Given two intervals $U,V\subset {\mathbb {R}}$ we define [*U*, *V*] to be the smallest interval containing both.

## Statement of Results

**Existence of infinitely renormalizable maps.** Our first task is to show that the class ${\mathcal {A}}(2^\infty )$ is non-empty. In other words, we need to establish strongly asymmetric maps with Feigenbaum–Coullet–Tresser dynamics. For maps which are differentiable at the extremal point, this follows from an analysis how kneading sequences depend on the parameter, see [51] or from some fixed point argument [46]. When $1=\alpha <\beta$ these proofs break down. In fact, if $\alpha =\beta =1$ holds (this corresponds to a family of tent maps) then there are no Feigenbaum–Coullet–Tresser maps.

Nevertheless we have the following theorem, showing that every family such as the one defined in (4) contains a map in ${\mathcal {A}}(2^\infty )$.

### Theorem 1

For the family defined in (4) there exists a parameter $t_*$ so that $f_{t_*}\in {\mathcal {A}}(2^\infty )$.

In fact, the proof of this theorem will show that any family similar to (4) (not necessarily with $\alpha =1$) is *full* in the sense that for each parameter *t* there exists $t_*$ so that $f_{t_*}$ has the same kneading invariant as $Q_t(x)=tx(1-x)$.

**The issue of real bounds** Since the power laws of *f* at both sides of 0 are different, most proofs from the theory of one-dimensional dynamics do not apply. The stumbling block appears already when trying to recover real bounds. For example, for ‘symmetric’ unimodal maps for which the power laws on both sides of 0 are the same, one has the property that the first entry map from the critical value *f*(0) to the interval $[a_n,b_n]$ has bounded distortion, see [46]. This kind of bound forms the cornerstone for everything else in the theory of unimodal maps, and so this is the first issue to overcome. In the weakly symmetric unimodal case the standard proof of such a real bound relies on the simple but powerful *smallest interval argument*, see Lemma 3. In the weakly symmetric case this argument gives space on both sides of some interval, and in the strongly asymmetric case only on one side, which prevents Koebe like distortion results. It turns out that this is not just a technical issue as the most basic real bounds do not hold. Indeed, the first entry map from the critical value into a periodic renormalization interval around the critical point does NOT have a diffeomorphic extension with Koebe space, see for example Theorem 2 below. Moreover, entirely new scaling phenomena appear as a result of this asymmetry.

The purpose of this paper is to make a step towards a theory for strongly asymmetric maps obtaining results on real bounds, scaling laws and absence of wandering intervals in this setting. Indeed we believe that the results described in this paper go through for all maps in ${\mathcal {A}}_{\alpha ,\beta }$ with $1\le \alpha <\beta$, although we were only able to do this under the assumption that $\alpha =1$. For the case that $1=\alpha <\beta$ we were able to exploit the almost linearity of the left branch near the turning point $c=0$, but when $1\le \alpha <\beta$ one should be able to exploit the huge asymmetry to obtain good control on the first entry maps. This is certainly what numerical simulations seem to suggest.

**No diffeomorphic extensions** The main source of difficulties lies in the following theorem, which shows the difference with the ‘symmetric’ case:

### Theorem 2

For every $\tau >0$ there exists $k_0\ge 0$ so that if $T\ni f(0)$ is the maximal interval on which $f^{2^k -1}|T$ is diffeomorphic, then $f^{2^k-1}(T)$ does **not** contain a $\tau$-scaled neighbourhood of $[a_k,b_k]$ for any $k\ge k_0$.

**Semi-extensions.** To overcome this issue, we will introduce the notion of semi-extension. Since $\alpha =1$, the derivative of *f* near the critical point of the left branch of *f* is non-zero and we can extend this branch smoothly ($C^3$) and monotonically to $f_1 : [a_0,\epsilon _0] \rightarrow {\mathbb {R}}$ in such a way that $\epsilon _0>0$, $f_1 |[a_0 ,0] = f$, the derivative of $f_1$ is strictly positive, and the Schwarzian derivative of $f_1$ is $\le 0$. For consistency, the right branch of *f* will be denoted by $f_2$, i.e. $f_2 = f |[0,b_0]$.

### Definition

(Semi-extensions). Let *J* be an interval and $f^n |J$ be monotone. Then $F : T\rightarrow {\mathbb {R}}$ is called *monotonic semi-extension* of $f^n |J$ if

- $$J\subset T$$ and $$F|J =f^n|J$$;
- $$F=f_{i_1} \circ \dots \circ f_{i_n},$$ where $$i_k\in \{1,2\}$$ for $$k=1,...,n$$.

We will call such an extension *maximal* if *T* is the maximal interval satisfying the above properties.

**Big bounds for the first entry maps to** $[a_k,b_k]$**when** *k* **is even.** It turns out that these semi-extensions are surprisingly useful since the branch $f_1$ is essentially linear near 0. Indeed, the semi-extension of the first entry map from an interval $J\ni f(0)$ to $[a_k,b_k]$ becomes almost linear for $k\rightarrow \infty$ and even. On the other hand, it turns out that as *k* odd and $k\rightarrow \infty$ this first entry map does **not** converge to a linear map.

### Theorem 3

Let $f^{2^k-1} :J\rightarrow [a_k,b_k]$ be the first entry map of $J\ni f(0)$ into $[a_k,b_k]$ and let $F_k :T_k \rightarrow {\mathbb {R}}$ be the maximal monotonic semi-extension of $f^{2^k-1} :J\rightarrow [a_k,b_k]$. Take $\tau _k>0$ be maximal so that $F_k(T_k)$ is $\tau _k$-scaled neighbourhood of $[a_k,b_k]$. Then

- $\lim \tau _{2k-1}= \lambda$ where $\lambda \in (0,1)$ is the root of the equation $\lambda ^\beta +\lambda =1$.
- $\tau _{2k}\approx b_{2k}^{-1/2}$ grows super-exponentially with *k*. In fact, $\log \tau _{2k}$ grows exponentially, see also Eq. (9) below.

### Remark 1

As we will show in Theorem 9 and Sect. 11, this theorem does not hold when we drop the assumption that $J\ni f(0)$. This will complicate for example the proof of Theorem 10 (on absence of wandering intervals).

**Scaling laws.** From this theorem we will obtain that the geometry of the $\omega$-limit set is quite different from the one found in smooth unimodal maps with $2^\infty$ combinatorics. In the next theorem we describe this scaling. By definition $f(a_k) = f(b_k)$ and therefore

5 $$\begin{aligned} a_k \sim - K_0b_k^\beta , \text{ where } K_0 = K_+/K_-. \end{aligned}$$

Thus the scaling properties of the renormalization intervals can be described just by the scaling properties of $b_k$.

### Theorem 4

The following scaling properties hold for $b_k$:

- For large even values of *k* one has
  6 $$\begin{aligned} b_{k+1}\sim & {} \lambda b_k \nonumber \\ c_{2^k}\sim & {} b_k, \end{aligned}$$
  where as before $\lambda \in (0,1)$ is the root of the equation $\lambda ^\beta +\lambda =1$.
- For large odd values of *k* one has
  7 $$\begin{aligned} b_{k+1}\sim & {} \beta ^{\frac{-2}{\beta -1}} K_0^{\frac{1}{\beta -1}} \lambda ^{-2} b_{k}^2 \nonumber \\ c_{2^{k}}\sim & {} -\beta ^{-\frac{\beta +1}{\beta -1}} K_0^{\frac{\beta }{\beta -1}} \lambda ^{-\beta -1} b_{k}^{\beta +1} \end{aligned}$$
- The length of the renormalization intervals decays super-exponentially fast: there exists $\Theta >0$ so that
  8 $$\begin{aligned} \log \left( \frac{1}{b_{2k}}\right) \sim \log \left( \frac{1}{|b_{2k}-a_{2k} |}\right) \sim \Theta \cdot 2^k . \end{aligned}$$
  More precisely,
  9 $$\begin{aligned} 1/b_{2k} \sim \beta ^{\frac{-2}{\beta -1}} K_0^{\frac{1}{\beta -1}} \exp (2^k \Theta ). \end{aligned}$$

In (6) the convergence is super-exponentially: $b_{k+1}/b_k$ converges to $\lambda$ super-exponentially fast.

**The parameter** $\Theta$**can be arbitrarily large.** The parameter $\Theta$ is determined by the asymptotic behaviour of $1/b_{2k}$. In the next corollary we show that $\Theta$ indeed varies within the space ${\mathcal {A}}(2^\infty )$:

### Corollary 1

For each $\Theta _0>0$ there exists a map $f\in {\mathcal {A}}_{1,\beta }(2^\infty )$ so that $\Theta (f)>\Theta _0$.

### Proof

From formula (9) it follows immediately that $\Theta (R^2(f))=2\cdot \Theta (f)$. $\quad \square$

**Renormalisation limits.** The above scaling laws make it possible to compute the renormalization map $R^k$ for *k* even with quite a lot of accuracy:

### Theorem 5

For *k* even we have

10 $$\begin{aligned} f^{2^k} (x) = {\left\{ \begin{array}{ll} c_{2^k} - s_k |x| +O(b_k^{\frac{3}{2}}) &{} \text{ when } x\in [a_k,0]\\ c_{2^k} - t_k x^\beta +O(b_k^{\frac{3}{2}}) &{} \text{ when } x\in [0,b_k] \end{array}\right. } \end{aligned}$$

where

11 $$\begin{aligned} s_k\sim \dfrac{b_k^{1-\beta }}{K_0} \text{ and } t_k\sim b_k^{1-\beta }. \end{aligned}$$

As usual we can state the renormalization results by rescaling the intervals to a fixed interval. So let $R^k f$ denote the *k*-th renormalization of *f*. In other words, let $l_k : [0, 1] \rightarrow [a_k , b_k ]$ be the linear map such that $l(0) = a_k$ and $l(1) = b_k$ and define $R^kf:=l_k^{-1} \circ f ^{2^k}\circ l_k$. Let ${{\hat{c}}}_k$ denote the the critical point of $R^k f$. From (5) it is clear that ${{\hat{c}}}_k \rightarrow 0$ as $k \rightarrow \infty$. Therefore, the left branch of $R^k f$ gets more and more degenerate and disappears in the limit.

### Theorem 6

The right branch of the renormalizations of *f* converge super exponentially fast in the $C^1$ norm to

$$\begin{aligned} \lim _{k\rightarrow \infty } (R^{2k} f )|[{{\hat{c}}}_k ,1]= & {} 1-x^\beta \\ \lim _{k\rightarrow \infty } (R^{2k+1}f )|[{{\hat{c}}}_k ,1]= & {} x^\beta . \end{aligned}$$

Let $m_k : [-1, 0] \rightarrow [0, {{\hat{c}}}_k ]$ be the linear orientation preserving maps mapping the boundary to the boundary. Then in the $C^1$ norm

$$\begin{aligned} \lim _{k\rightarrow \infty } (R^{2k} f ) \circ m_{2k}= & {} x+1\\ \lim _{k\rightarrow \infty } (R^{2k+1} f ) \circ m_{2k+1}= & {} -\lambda ^{\beta ^2-1} (x+\lambda ^{-\beta })^\beta + \lambda ^{-1}. \end{aligned}$$

Here the convergence is super exponentially fast as well and $\lambda \in (0, 1)$ is the root of $\lambda ^\beta + \lambda = 1$ as before.

It is easy to see that $\lambda ^\beta + \lambda = 1$ implies that $-\lambda ^{\beta ^2-1} (x+\lambda ^{-\beta })^\beta + \lambda ^{-1}$ is equal to 1 when $x=-1$ and equal to 0 when $x=0$. Note that the asymptotic expression for the left branch of $R^{2k+1}f$ is an explicit but non-trivial expression.

### Remark 2

One can prove also convergence in the $C^N$ norm in the above theorem if *f* is a smooth function outside of zero. If the map *f* is only assumed to have finite smoothness this can be done as in [26] or following the approach in [5]. If *f* is real analytic (on each side of 0) then this can be done by complex tools: then $f^{2^k}=E_k\circ f$ where $E_k$ extends holomorphically to a diffeomorphism whose range is $B(0,\tau _k|b_k|)$. Using the Koebe Lemma (in the complex case) we then obtain that, for *k* even, $DE_k=DE_k(c_1) + o(k)$ and $D^iE_k=o_i(k)$ for each $i\ge 2$. The speeds of convergence can be obtained from Koebe and from the speed of $\tau _k$.

**Metric invariants and universality.** Theorem 4 implies that two maps $f,{{\tilde{f}}}\in {\mathcal {A}}(2^\infty )$ are not necessarily differentiably conjugate on their postcritical sets. In fact, there are necessary and sufficient conditions which are needed for universality:

### Theorem 7

(Complete invariants for $C^1$ universality). Take two maps $f\in {\mathcal {A}}_{1,\beta }(2^\infty )$ and $\tilde{f}\in {\mathcal {A}}_{1,{{\tilde{\beta }}}}(2^\infty )$, with as before $\beta ,{{\tilde{\beta }}}>1$. Then there exists a homeomorphism *h* which is a conjugacy between the postcritical sets of $f,{{\tilde{f}}}$ and

1. *h* is Hölder at 0;
2. *h* is Lipschitz at 0 $$\iff $$*h* is differentiable at 0 $$ \iff $$$$\Theta ={{\tilde{\Theta }}}$$ and $$\beta ={{\tilde{\beta }}}$$.

Here $\Theta$ is defined through Eq. (8) in Theorem 4.

Moreover, let $\Lambda =\overline{\cup _n f^n(0)}$ be the attracting Cantor set and ${{\tilde{\Lambda }}}$ be the corresponding set for $\tilde{f}$. Then $\Theta ={{\tilde{\Theta }}}$ and $\beta ={{\tilde{\beta }}}$ implies that the conjugacy $h:\Lambda \rightarrow {{\tilde{\Lambda }}}$ is differentiable in the sense that the following limit exists

$$\begin{aligned} \lim _{y\in \Lambda , y\rightarrow x} \dfrac{h(y)-h(x)}{y-x} \ne 0 \end{aligned}$$

and depends continuously on $x\in \Lambda$.

### Corollary 2

*f* and $R^2(f)$ are not Lipschitz conjugate.

### Proof

This follows from the previous theorem and Corollary 1. $\quad \square$

**Hausdorff dimension of the Attracting Cantor set.** As in the symmetric case the closure of the orbit of the critical point of $f\in {\mathcal {A}}(2^\infty )$ is a Cantor set which we denote as $\Lambda (f)$.

### Theorem 8

The Hausdorff dimension of the Cantor set $\Lambda (f)$, where $f \in {\mathcal {A}}(2^\infty )$, is zero.

**Absence of Koebe space.** In Theorem 3 we showed that there is a monotonic semi-extension of the branch of $f^{2^k-1}$ defined around the critical value with nice bounds. The next theorem shows that such a property does not hold for all points of the interval.

### Theorem 9

For each $\tau >0$ there exists *x* and *k* so that the maximal semi-extension of the first entry map of *f* from *x* into $[a_k,b_k]$ does **not** contain a $\tau$-scaled neighbourhood of $[a_k,b_k]$.

**Absence of wandering intervals.** As usually, one says that *W* is a wandering interval if all iterates of *W* are disjoint and if *W* is not in the basin of a periodic attractor. Existing proofs for absence of wandering intervals do not go through. Indeed, we used an argument which is quite different from anything we have seen in the literature showing that

### Theorem 10

No map $f\in {\mathcal {A}}_{1,\beta }(2^\infty )$ has wandering intervals.

## Some Background Material

In the proofs below we will need the well-known *Koebe Theorem*.

### Lemma 1

(Koebe Lemma) Let $g:T\rightarrow g(T)$ be a $C^3$ diffeomorphism with $Sg<0$. Assume that $J\subset T$ is an interval so that *g*(*T*) contains a $\tau$-scaled neighbourhood of *g*(*J*), i.e. $g(T)\supset (1+\tau ) g(J)$. Then for all $x,y\in J$,

$$\begin{aligned} \dfrac{\tau ^2}{(1+\tau )^2} \le \dfrac{Dg(x)}{Dg(y)} \le \dfrac{(1+\tau )^2}{\tau ^2} \end{aligned}$$

and

$$\begin{aligned} \dfrac{\tau }{1+\tau } \dfrac{|g(J)|}{|J|} \le |Dg(y)| \le \dfrac{1+\tau }{\tau } \dfrac{|g(J)|}{|J|}. \end{aligned}$$

### Proof

See the proof of Theorem IV.1.2 in [47]. $\quad \square$

Integrating the last inequalities immediately gives:

### Lemma 2

(Corollary of Koebe). Let *g* be as in the previous lemma and let $L:J\rightarrow g(J)$ be the affine surjective map with the same orientation as *g*. Then for all $x\in J$,

$$\begin{aligned} Lx - \dfrac{1}{1+\tau } |g(J)| \le g(x)\le L x + \dfrac{1}{\tau } |g(J)|, \quad | \dfrac{Dg(x)}{DL(x)}-1| \le \dfrac{1}{\tau } . \end{aligned}$$

## Unusual Bifurcations of Families of Maps with Strong Asymmetries

In this section we will consider the local bifurcation of families of maps $g_t$ with strong asymmetries. For simplicity, take $\beta>1,A>1$ and let us consider a concrete example:

$$\begin{aligned} g_t(x)=\left\{ \begin{array}{rl} A|x|+t &{} \text{ for } x\le 0\\ x^\beta +t&{} \text{ for } x\ge 0.\end{array}\right. \end{aligned}$$

**Fig. 3:**
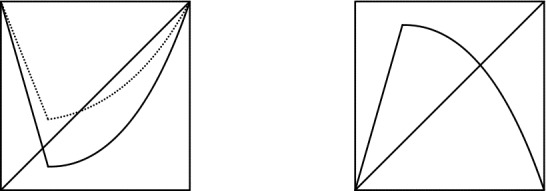
$f^{2^n}|I_{n,t}$ for *n* odd (on the left) and *n* even (on the right). When $n\ge 2$ is even then $I_{n,t}\rightarrow \{0\}$ as $t\downarrow u_n$ and for $t\in (u_n,v_n)$ the only fixed point of $f_t^{2^n}$ in the interior of $I_{n,t}$ lies to the right of 0

For $t>0$ this maps has an attracting fixed point, whereas for *any* $t<0$ near 0 this has a repelling fixed point *p*(*t*) and an attracting periodic orbit $\{q_1(t),q_2(t)\}$ with period 2 with $q_1(t)<p(t)<0<q_2(t)$, see the left panel of Fig. 3. So periodic doubling occurs precisely when 0 is a fixed point of $g_t$ . We will call this an asymmetric period doubling bifurcation.

Note that if we take a map with the opposite orientation, say ${{\hat{g}}}_t(x)=-g_t(x)$, then the attracting fixed point disappears as soon as $t<0$ (so this is the analogue of the saddle-node bifurcation).

In the next section we will consider the analogue of the periodic doubling phenomena for a family of maps $f_t$ in $\mathcal A_{1,\beta }$. During this parameter window only period doubling occurs. The usual period doubling occurs when an attracting periodic orbit of period $2^{2n}$ becomes repelling and creates an attracting periodic orbit of period $2^{2n+1}$ (when the multiplier is equal to $-1$). On the other hand, the asymmetric periodic doubling occurs when an attracting periodic orbit of period $2^{2n+1}$ looses stability as it goes through the turning point 0.

## The Existence of a $2^\infty$ Map Within the Space of One-Sided Linear Unimodal Maps and a Full Family Result

This section is the only one in this paper where we consider maps in ${\mathcal {A}}_{\alpha ,\beta }$ where we allow $\alpha \ge 1$. In fact, in the proof of Theorem 11 below we assume $\alpha =1$, because when $\alpha >1$ the proof is simpler: in that case the proofs in [51] (for the unimodal setting) and in [46] (for the multimodal setting) go through.

We say that a non degenerate interval *I* is *restrictive* of period $d>0$ of a unimodal map *f* if it contains the critical point of *f*, the interiors of $I, f(I), \ldots , f^{d-1}(I)$ are disjoint and $f^d(I)\subset I$, $f^d (\partial I) \subset \partial I$. If a map *f* has a restrictive interval *I* of period *d* is called *renormalizable* and $f^d|I$ is called a renormalization of *f*. Note that any renormalization of a unimodal map is unimodal.

The maps in class ${\mathcal {A}}_{\alpha ,\beta }(2^\infty )$ we defined are all infinitely renormalizable, moreover all the restrictive intervals $I_1 \supset I_2 \cdots \supset I_n \cdots$ are of periods $2,2^2,\ldots ,2^n,\ldots$.

The following theorem implies Theorem 1:

### Theorem 11

Consider a family $f_t:[a_0,b_0]$, $t\in [0,1]$ in $\mathcal A_{\alpha ,\beta }$ with $1\le \alpha <\beta$ so that $t\mapsto f_t|[a_0,0] \in C^1$ and $t\mapsto f_t|[0,b_0] \in C^1$ are continuous and so that $f_0$ has a unique attracting fixed point and so that $f_1$ is surjective. Then there exist two sequences of parameters $u_1<u_2<\cdots<v_2<v_1$ such that

- for $t \in (u_n, v_n]$ the map $f_t$ is $2^n$ renormalizable, more precisely, there exists a non degenerate restrictive interval $I_{n,t}$ of period 2 of the map $f_t^{2^{n-1}}|I_{n-1,t}$ continuously depending on the parameter $t\in (u_n, v_n]$ (here we set $I_{0,t}=[a_0,b_0]$);
- when *n* is even then $f_{u_n}^{2^{n-1}}(0)=0$ and $\lim _{t\downarrow u_n} I_{n,t}=\{0\}$, while for *n* is odd $f_{u_n}$ has a parabolic periodic orbit of period $2^{n-1}$ with multiplier $-1$ and and $\lim _{t\downarrow u_n} I_{n,t}$ is non-degenerate;
- $f_{v_n}^{2^n}(I_{n,v_n}) = I_{n,v_n}$, that is $f_{v_n}^{2^n}|I_{n,v_n}$ is surjective.

Clearly, $f_t \in {\mathcal {A}}_{\alpha ,\beta }$ for any $t \in \cap _n (u_n,v_n)$.

Note that $\cap _n (u_n,v_n)\ne \emptyset$ because the intervals $(u_n,v_n)$ are properly nested. In particular, the family (4) (with $\beta >1$) contains a map in the class ${\mathcal {A}}_{\alpha ,\beta }(2^\infty )$.

### Proof

The proof we will give of this theorem is almost the same as a proof based on a bifurcation analysis for smooth unimodal maps and will use the following two properties:

(1) whenever $f_t$ has an attracting periodic orbit then 0 is in the immediate basin of this attractor. This holds since *f* has negative Schwarzian derivative, and therefore the immediate basin of a periodic attractor contains a turning point of an iterate of *f* and hence 0 is also in the immediate basin of this periodic attractor.

(2) whenever 0 is a (topologically) *attracting* periodic point of $f_{t_0}$ of period *n* then $f_t$ has a periodic attractor of period *n* or period 2*n* for each *t* near $t_0$. Note that within this class of maps it is no longer true that if 0 is periodic then it is also attracting (it can be repelling on one side when $\alpha =1$).

Analysing what bifurcations occur in the family $f_t$ analogous to the period doubling bifurcations which occur in the quadratic family, we will prove inductively that there exists a nested sequence of maximal parameter intervals described by the theorem.

Slightly abusing notation we set $u_0=0$, $v_0=1$ and $I_{0,t}=[a_0,b_0]$. Clearly all the properties stated in the theorem are satisfied except one claiming that the critical point is fixed by $f_0$. This does not affect the proof which follows. So assume by induction that such parameter interval $[u_n,v_n]$ exists for some integer *n*. There are two possibilities.

(i) *n* is even. In this case for each $t\in [u_n,v_n]$, $f_t^{2^n}|I_{n,t}$ is of type $+-$ and $\alpha \beta$, i.e., orientation preserving (resp. reversing) to the left (right) of 0 and the order of the critical point is of order $\alpha$ to the left of 0 and of order $\beta$ to the right of 0. We know that $f_{v_n}^{2^n}|I_{n,v_n}=I_{n,v_n}$, therefore there exists an orientation reversing fixed point $p_n>0$ of $f_{v_n}^{2^n}|I_{n,v_n}$. Note that this fixed point is repelling because the orbit of the critical point of $f_{v_n}^{2^n}$ belongs to the boundary of $I_{n,v_n}$. Since the multiplier of $p_n$ is not equal to one this fixed point persists when we change a parameter in a neighbourhood of $v_n$, that is there is a continuous function $p_{n,t}$ defined for *t* in some interval $W_n\ni v_n$ such that $f^{2^n}_t(p_{n,t})=p_{n,t}$ and $p_{n,v_n}=p_n$. We will assume that $W_n$ is the maximal interval where such a function can be defined. Let $u_{n+1}< v_n$ be maximal such that $Df_{u_{n+1}}^{2^n}(p_{n,u_{n+1}})=-1$, that is $p_{n,u_{n+1}}$ becomes a parabolic periodic point of *f* with multiplier $-1$. Such a point $u_{n+1}$ exists and $u_{n+1}>u_n$ because the multiplier of $p_{n,t}$ varies continuously with the parameter $t\in W_n\cap (u_n,v_n]$, since $Df^{2^n}_t(p_{n,t})<-1$ for $t=v_n$ and since for any *t* we have $\lim _{x\downarrow 0} Df^{2^n}_t(x)=0$ while $f_{u_n}^{2^{n-1}}(0)=0$.

For $t\in [u_{n+1}, v_n]$ let ${{\hat{p}}}_{n,t}<0$ denote a preimage of $p_{n,t}$ under $f_t^{2^n}|I_{n,t}$ and let $I_{n+1, t}=[{{\hat{p}}}_{n,t}, p_{n,t}]$. Since *f* has negative Schwarzian derivative it follows that $p_{n,u_{n+1}}$ is a parabolic periodic point of $f_{u_{n+1}}$ and that the critical point belongs to the basin of attraction of $p_{n,u_{n+1}}$. This in turn implies that $f_{u_{n+1}}^{2^{n+1}}(I_{n+1,u_{n+1}}) \subset I_{n+1,u_{n+1}}$, i.e., $I_{n+1,u_{n+1}}$ is a restrictive interval of $f_{u_{n+1}}^{2^n}$ of period 2. Note that if *t* is slightly larger than $u_{n+1}$, the interval $I_{n+1,t}$ is still a restrictive interval of period 2 of the corresponding map. We know that $f_{v_n}^{2^n}(0)$ belongs to the boundary of $I_{n,v_n}$ and therefore $f_{v_{n}}^{2^{n+1}}(0) \not \in I_{n+1, v_n}$. Define $v_{n+1}$ to be infimum of all parameters $t > u_{n+1}$ such that $f_{v_{n+1}}^{2^{n+1}}(0) \not \in I_{n+1, v_n}$, thus $f_{v_{n+1}}^{2^{n+1}}(0)$ belong to the boundary of $I_{n+1,v_{n+1}}$. It must be the left boundary point (that is $f_{v_{n+1}}^{2^{n+1}}(0) = {{\hat{p}}}_{n,v_{n+1}}$) because otherwise the condition $Df_t^{2^n}(p_{n,t}) \le -1$ for $t\in [u_{n+1},v_n]$ would be broken.

It is easy to see that the constructed points $u_{n+1}$, $v_{n+1}$ and the intervals $I_{n+1, t}$ satisfy all the induction assumptions. Note that in this case the intervals $I_{n+1, t}$ are non degenerate for all $t \in [u_{n+1}, v_{n+1}]$.

(ii) *n* is odd. In this case $$ f_{u_n}^{2^n}|I_n$$ is of type $$-+$$ and $$\alpha \beta $$. The construction will be very similar to the case of even *n* with some modifications relating to the asymmetric period doubling bifurcation.

Arguments similar to the case when *n* is even show that there exists a maximal $u_{n+1} < v_n$ such that $f_{u_{n+1}}^{2^n}(0)=0$. Then for all $t\in [u_{n+1}, v_n]$ there exists an orientation reversing fixed point $p_{n,t} \in I_{n,t}$ of $f_t^{2^n}$. Note that $p_{n,t}$ is negative (i.e. it is to the left of the critical point). Define ${{\hat{p}}}_{n,t}>0$ to be a preimage of $p_{n,t}$ under $f_t^{2^n}|I_{n,t}$ and let $I_{n+1, t}=[{{\hat{p}}}_{n,t}, p_{n,t}]$ for all $t\in [u_{n+1}, v_n]$ as before. Note that $p_{n,u_{n+1}}={{\hat{p}}}_{n,u_{n+1}}=0$ and the interval $I_{n+1, u_{n+1}}$ degenerates to the critical point. For all other values of the parameters the intervals $I_{n+1, t}$ are non degenerate. In Sect. 5 it was explained that for values of parameters *t* slightly larger than $u_{n+1}$ the interval $I_{n+1,t}$ is a restrictive interval of period 2 of the map $f_t^{2^n}$. As before define $v_{n+1}> u_{n+1}$ to be maximal such that $I_{n+1,t}$ is a restrictive interval of period 2 of the map $f_t^{2^n}$ for all $t\in (u_{n+1}, v_{n+1})$ and note that $v_{n+1} < v_n$. $\quad \square$

In fact, we have

### Theorem 12

Any family $\{f_t\}$ as in Theorem 11 is a full family in the following sense. Take a quadratic interval map *Q* without periodic attractors. Then there exists a parameter *t* so that $f_t$ combinatorially equivalent to *Q*.

### Proof

In [51], see also [20], this result is shown for families $f_t$ of unimodal maps with $\alpha ,\beta >1$. Let us give an outline of that proof. The main ingredients are the notion of the *kneading invariant* $\nu (f)$ of a unimodal map *f*, the abstract notion of an *admissible kneading sequence* $\nu$, the lexicographical ordering on the space of kneadings, and a topology on this space. The required result follows by showing that for each admissible kneading sequence $\nu$ there exists *t* so that $\nu =\nu (f_t)$. Proving this relies on some kind of intermediate value in the space of kneadings, by analysing the discontinuities of the map $t\mapsto \nu (f_t)$ and using the following two observations:

1. if $t_0$ is a parameter for which the critical point of $f_{t_0}$ is non-periodic, then the kneading invariant $t\mapsto \nu (f_t)$ is continuous at $t=t_0$;
2. if $t_0$ is a parameter for which the critical point of $f_{t_0}$ is periodic, then for $t\approx t_0$ the map $f_t$ still has a periodic attractor (here it used that $\alpha ,\beta >1$). This then makes it possible to show that for each $s,t\approx t_0$ the kneading sequences $\nu (f_t)$ and $\nu (f_s)$ are the same up to a simple operation (related to some star product). Thus one obtains that there are no admissible kneading sequences that get skipped.

In our case, when $\alpha =1<\beta$ the first step still holds, but in the 2nd step the map $f_t$ may not have a periodic attractor when $t\approx t_0$. However, as is shown in the previous theorem, the kneading sequences for nearby maps still bifurcate the same way as they do for nearby smooth maps. Thus the proof in [51], see also [20], goes through.

Another way of proving this theorem is by adapting the proof given in  [46, Theorem II.IV.1]. That proof follows a Thurston mapping approach and, contrary to the proof from [51], also applies to multimodal families. To apply this proof in our setting, one needs to show that a certain map defined on some open symplex is ‘repelling’ near the boundary of this simplex. We will not give the details for the required modifications here. $\quad \square$

## The Smallest Interval Argument

The usual smallest interval argument in the current setting gives a weaker statement than in the ‘symmetric’ case:

### Lemma 3

There exists $\tau >1$ so that the following holds. Consider $I=[a_n,b_n]$ and choose $x\notin I$. Assume that there exists $k>0$ (minimal) so that $f^k(x)\subset I$. Then there exists an interval $T\ni x$ so that $f^k|T$ is a diffeomorphism and $f^k(T)\supset [\tau a_n,\tau b_n]$.

### Proof

For completeness let us include the proof of this lemma. Let *T* be the maximal interval $T\ni x$ so that $f^k|T$ is a diffeomorphism. By maximality of *T* and since $f^i(x)\notin I$ for all $i=0,\dots ,k-1$ there exist integers $0<i_0,i_1<2^n$ so that $f^k(T)\supset [f^{i_0}(I),f^{i_1}(I)]$ where $f^{i_0}(I)$ and $f^{i_1}(I)$ are to the left respectively to the right of *I*. So it suffices to show that $[f^{i_0}(I),f^{i_1}(I)]\supset [\tau a_n,\tau b_n]$ for some universal choice of $\tau >0$.

Write $I_i=f^i(I)$ and let $3\le m\le 2^n$ be so that $I_m$ is the smallest of the intervals $I_3,\dots ,I_{2^n}$. Let $K_m$ be the smallest interval containing the left and right neighbours of $I_m$ from the collection $I_1,\dots ,I_{2^n}$ (such neighbouring intervals exist because $m\ge 3$). It follows that $K_m$ contains a $\tau _0$-scaled neighbourhood of $I_m$ where $\tau _0>0$ is independent on *n* (here we use that $I_1,I_2$ are not much smaller than $I_3$). Let $K_1\supset I_1$ be the maximal interval on which $f^{i_0-1}|K_1$ is a diffeomorphism with $f^{i_0-1}(K_1)\subset K_m$. By maximality, $f^{i_0-1}(K_1)= K_m$. By Koebe it follows that $K_1$ contains a $\tau _1$-scaled neighbourhood of $I_1$. Hence $K_0:=f^{-1}(K_1)$ contains $[\tau _1' a_n,\tau _1'' b_n']$ where $\tau _1'=\tau _1^{1/\alpha }$ and $\tau _1''=\tau _1^{1/\beta }$. Note that because $|a_n|<<b_n$, this latter interval is no longer a definite interval around $[a_n,b_n]$. Note also that by the choice of $K_m$ the interval $K_0$ is contained in any interval of the form $[f^{i_0}(I),f^{i_1}(I)]$ where $f^{i_0}(I)$ and $f^{i_1}(I)$ are to the left respectively to the right of *I*. $\quad \square$

## Big Bounds

Since $\alpha =1$, we can consider a semi-extension of *f* of the ‘linear’ branch and use the following strategy. First, using the standard smallest interval argument we have already shown that there exists a definite space to the right of the renormalization intervals. Next we will show that either there is definite space to the left of the renormalization interval for the semi-extension or this space is at least as big as the space on the previous level. Considering several scenarios, this will imply that there is some definite space on both sides of the renormalization intervals (for the semi-extension). Once there is ‘space’ on both sides of the renormalization intervals we can repeat the argument used to obtain it and get as much space as one may want. From this the rest follows.

### Using semi-extensions

Let $f^{2^{k}-1} :J_k \rightarrow [a_k , b_k ]$ be the branch of the first entry map to $[a_k , b_k ]$ for which $c_1 := f (0) \in J_k$. Note that this is a surjective diffeomorphism. Let ${{\hat{T}}}_k\supset J_k$ be the maximal interval around *f*(0) so that $f^{2^{k}-1}|{{\hat{T}}}_k$ is a diffeomorphism and let $[{{\hat{A}}}_k,{{\hat{B}}}_k]:=f^{2^{k}-1}({{\hat{T}}}_k)$ where ${{\hat{A}}}_k<{{\hat{B}}}_k$. Note that $f^{2^{k}-1}|{{\hat{T}}}_k$ is orientation preserving (reversing) when *k* is even (odd). We also define an interval $[A_k,B_k]\supset [{{\hat{A}}}_k,{{\hat{B}}}_k]$, with $A_k<B_k$, associated to the semi-extension as follows. Let $E_k : T_k\rightarrow [A_k , B_k ]$ be the maximal monotone surjective *semi-extension* of $f^{2^{k}-1}:J_k \rightarrow [a_k , b_k ]$ such that $A_k\le a_k< 0 < b_k \le B_k$. (In principle this extension depends on the choice of the extension $f_1:[0,\epsilon )\rightarrow {\mathbb {R}}$ of $f:[a_0,0]\rightarrow {\mathbb {R}}$.)

### Useful dynamical and non-dynamical points $a'_k,b_k',d_k,e_k$.

**Fig. 4:**
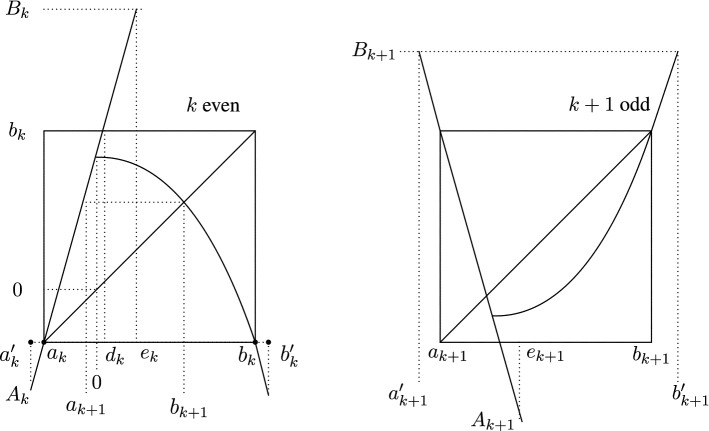
$f^{2^k}|I_k$ and $f^{2^{k+1}}|I_{k+1}$ when *k* is even and their semi-extensions. Note that the points $d_k,e_k,a_k',b_k'$ are defined using the semi-extension rather than dynamically

Let $[a'_k , e_k ] = f_1 ^{-1}(T_k )$, $a_k'<a_k<0<e_k$, and therefore $E_k \circ f_1 : [a'_k , e_k ] \rightarrow [A_k , B_k ]$ is the maximal monotone surjective semi-extension of $f^{2^k} : [a_k , 0] \rightarrow [a_k , b_k ]$. Also, define the point $b'_k > b_k$ as the right boundary point of the interval $f_2^{-1}(T_k )$. Furthermore, define $d_k \in [0, e_k ]$ such that $E_k \circ f_1 (d_k ) = b_k$ for even values of *k*. When *k* is odd the point $d_k$ is not defined. The properties of these points are made clear in Fig. 4 and the purpose of these points is expanded on in Sect. 8.3 where a sketch of the proof of Theorem 3 is given.

Since $E_k$ is orientation preserving (reversing) when *k* is even (odd), the following holds:

- for even values of *k*
  $$\begin{aligned} A_k= & {} E_k \circ f_1 (a'_k ) = E_k \circ f_2 (b'_k ), \\ B_k= & {} E_k \circ f_1 (e_k ) \end{aligned}$$
- and for odd *k*
  $$\begin{aligned} B_k= & {} E_k \circ f_1 (a'_k ) = E_k \circ f_2 (b'_k ),\\ A_k= & {} E_k \circ f_1 (e_k ). \end{aligned}$$

As we will show in Lemma 4, $B_k={{\hat{B}}}_k$ but in general $A_k\ne {{\hat{A}}}_k$.

### Sketch of the proof of Theorem 3

**Fig. 5:**
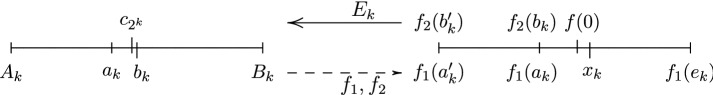
When *k* is even, $E_{k+1}=E_k\circ f_2 \circ E_k$ and $E_k$ is orientation preserving. Here $E_k(x_k)=b_k$. It is not clear where $b_k'$ and $a_k'$ are in relation to $B_k$ and $A_k$

Note that the interval $[A_k,B_k]$ is the range of the *semi-extension* of the first entry map $E_k$ (rather than its diffeomorphic extension), see Fig. 5. Therefore none of the points $A_k,B_k,a_k',b_k',e_k$ have a priori any dynamical interpretation. As it turns out $B_k={{\hat{B}}}_k$, see Lemma 4 and therefore $B_k$ has a dynamical interpretation, but none of these other points do.

Our aim in this section is to show $[A_k,B_k]$ is much bigger than $[a_k,b_k]$ (for *k* even and large). To do this, we will consider all the various positions of $a_k',b_k',e_k$ and show that each of these give some recursive information. Let us outline the argument.

**Step 1** (Sect. 8.4) consists in obtaining various topological properties, including that if $e_{k+1}<b_{k+1}$ then one can propagate the semi-extension of level $k+1$ to level $k+2$. More precisely, for *k* even

12 $$\begin{aligned} e_{k+1}<b_{k+1} \implies A_{k+2}=A_{k+1}. \end{aligned}$$

**Step 2** (Sect. 8.5) consists in using some cross-ratio inequality and the strong-asymmetry of *f* to show that there exists a $C>0$ so that the following recursive inequality holds for all even *k*

13 $$\begin{aligned} d_k \le C b_{k+1}^{\beta -1} b_k. \end{aligned}$$

**Step 3** (Sect. 8.6) gives the following dichotomy, see Lemma 7,

14 $$\begin{aligned} \text{ either } |A_k | > C b_{k+1} \text{ or } e_k < b_{k+1}. \end{aligned}$$

**Step 4 ** (Sect. 8.7) shows that the assumption $|A_k|>C b_{k+1}$ implies some distortion control of the restriction of $f^{2^k}$ to $[b_{k+1},b_k]$, see Lemma 8.

**Step 5** (Sect. 8.8) consists in showing that one has infinitely often space. This means that we need to show that there exists $\tau >1$ so that $[A_k,B_k] \supset \tau [a_k,b_k]$ for infinitely many *k* even. From the smallest interval argument in Lemma 3 and the strong asymmetry we have that $B_k>\tau b_k>>a_k$ for some $\tau >1$. So it suffices to show that there exists $C>0$ so that $|A_k|>C b_{k}$ holds for infinitely many *k*. From the dichotomy (14) it follows that either from time to time the inequality $|A_k|>C b_{k+1}$ holds or $e_k<b_{k+1}$ holds for all *k* even and large. If the latter holds, then (12) implies that $A_{k+2}=A_{k+1}$ for *all* *k* even and large. Using a further argument using Eq. (13), using Step 4, we can then ‘replace’ the inequality $|A_k|>Cb_{k+1}$ by the inequality $|A_k|>Cb_k$, and obtain in Lemma 9 that

$$\begin{aligned} |A_k|>Cb_k \text{ holds } \text{ only } \text{ finitely } \text{ often } \implies \exists k_0 \text{ with } A_{k_0}=A_{k_0+1}=A_{k_0+2}=\dots . \end{aligned}$$

Of course the latter also implies $|A_k|>Cb_k$ for *k* large, thus concluding Step 5.

**Step 6** (Sect. 8.9) consists in showing that if $|A_k|>C b_k$ for some even *k* (or in other words if the space condition $[A_k,B_k] \supset \tau [a_k,b_k]$ holds) then one gets *large* space in the next step. Thus we obtain an increasingly growing space.

**Step 7** (Sect. 8.10) In this final step we show that the space is growing superexponentially fast. This is done in Lemma 12, and this then concludes the proof of Theorem 3.

### Some topological properties of $a'_k,b_k',d_k,e_k$

Let us list a number of more or less obvious relations between the points we defined. For example, assertion (4) and (5) show that if some metric properties hold for the non-dynamically defined points $b_k'$ and $e_k$ then the semi-extension from one level can be used to obtain a semi-extension of the next level.

#### Lemma 4

Let $k\ge 2$ be an even integer. Then

1. $$B_{k+1} = B_{k+2}={{\hat{B}}}_{k+1}={{\hat{B}}}_{k+2}=c_{2^k}$$;
2. $$e_{k+2 }< d_k$$;
3. $${{\hat{A}}}_{k}={{\hat{A}}}_{k+1}=c_{2^{k-1}}$$;
4. if $b'_k < B_k$, then $e_{k+1} < e_k$ and $A_{k+1}= A_k$.
5. if $e_{k+1} < b_{k+1}$, then $b'_{k+2} < b_{k+1}$ and $A_{k+2} = A_{k+1}$.

#### Proof

Since $$f^{2^k}[a_{k+1},b_{k+1}]\subset [0,b_k]$$, we have $$E_{k+1}=E_k \circ f_2\circ E_k|T_{k+1}$$, where $$E_k$$ is orientation preserving and $$f_2$$ is orientation reversing. Since the diffeomorphic range of $$E_k$$ is $$[{{\hat{A}}}_k,{{\hat{B}}}_k]\supset [a_k,b_k]\ni 0$$ and $$E_k\circ f_2$$ maps $$(0,b_k]$$ diffeomorphically onto $$[a_k,c_{2^k})$$, it follows that $$B_{k+1}={{\hat{B}}}_{k+1}=E_k\circ f_2(0)=c_{2^k}$$ and $$A_{k+1}\le {{\hat{A}}}_{k+1}\le a_k$$. Taking $$a_{k+1}'$$ to be the point in $$(a_k,a_{k+1})$$ for which $$f^{2^k}(a_{k+1}')=E_k\circ f_1(a_{k+1}')=0$$ one has $$f^{2^{k+1}}(a'_{k+1})=E_{k+1}\circ f_1(a'_{k+1})= E_k\circ f_2 \circ E_k\circ f_1(a_{k+1}')=E_k\circ f_2 \circ E_k(0)= B_{k+1}$$.

Similarly, since $f^{2^{k+1}}[a_{k+2},b_{k+2}]\subset [a_{k+1},0]$, $E_{k+2}=E_{k+1}\circ f_1\circ E_{k+1}|T_{k+2}$ where $E_{k+1}$ is orientation reversing and $f_1$ is orientation preserving. Since $a_k<a'_{k+1}<a_{k+1}<c_{2^{k+1}}=E_{k+1}(c_1)<0$, $E_{k+1}\circ f_1(a'_{k+1})=B_{k+1}={{\hat{B}}}_{k+1}$ and since the diffeomorphic range of $E_{k+1}$ is $[{{\hat{A}}}_{k+1},{{\hat{B}}}_{k+1})\supset [a_k,c_{2^k}) \supset (a_{k+1}',0)$ it follows that $B_{k+2}={{\hat{B}}}_{k+2}=B_{k+1}={{\hat{B}}}_{k+1}=c_{2^k}$ and ${{\hat{A}}}_{k+2}=c_{2^{k+1}}$, proving in particular statement (1).

By definition $E_{k+2}\circ f_1(e_{k+2})=B_{k+2}$. Since $E_{k+1}\circ f_1(a'_{k+1})=B_{k+1}=B_{k+2}$ and $E_{k+2}=E_{k+1}\circ f_1\circ E_{k+1}|T_{k+2}$ we have that $E_{k+1}\circ f_1(e_{k+2})=a'_{k+1}$. Since $a'_{k+1}\in (a_k,a_{k+1})$, $E_{k+1}\circ f_1(d_k)=E_k\circ f_2\circ E_k\circ f_1(d_k)=E_k\circ f_2 (b_k)=a_k$ and $E_{k+1}$ is orientation reversing, it follows that $e_{k+2}<d_k$, proving statement (2).

Statement (3) follows as in statement (1).

To prove statement (4), assume $b'_k < B_k$. Then $E_k$ has range $[A_k,B_k]\supset [A_k,b'_k]$. Note that the left endpoint of the domain of $E_k$ is $f_2(b'_k)$ and $E_k\circ f_2(b'_k)=A_k$. Since $E_{k+1}=E_k\circ f_2 \circ E_k$ it follows that the range of $E_{k+1}$ is equal to $[A_k,B_{k+1}]$ and so $A_{k+1}=A_k$. Moreover, $A_k=A_{k+1}=E_{k+1}\circ f_1(e_{k+1})=E_k\circ f_2 \circ E_k \circ f_1(e_{k+1})$ and $E_k\circ f_2(b'_k)=A_k$. Since $E_{k+1}$ and $f_1,f_2$ are all injective, $b'_k= E_k \circ f_1(e_{k+1})$. Therefore, and since $B_k=E_k\circ f_1(e_k)$ and $f_1,E_k$ are increasing, $b'_k<B_k$ implies that $e_{k+1}<e_k$.

Finally, to prove statement (5), note that $$E_{k+1}|[f(a_{k+1}),f(0))$$ maps diffeomorphically onto $$(c_{2^{k+1}}, b_{k+1}]$$ and if $$e_{k+1} < b_{k+1}$$ then this last interval contains $$(c_{2^{k+1}},e_{k+1}]$$. Since $$E_{k+1}\circ f_1$$ maps the latter interval diffeomorphically onto $$[A_{k+1},c_{2^{k+2}})$$ and since $$E_{k+2}=E_{k+1}\circ f_1 \circ E_{k+1}|T_{k+2}$$ it follows that $$A_{k+2}= A_{k+1}$$ and $$b'_{k+2} = f^{2^{k+1}} |[0,b_{k+1}](e_{k+1}) < b_{k+1}$$. $$\quad \square $$

### A first recursive inequality

#### Lemma 5

There exists $C>0$ so that for all *k* even

15 $$\begin{aligned} d_k \le C b_{k+1}^{\beta -1} b_k. \end{aligned}$$

#### Proof

For *k* even, $b_{k+1}$ is a repelling fixed point of $f^{2^k}$, so $|Df^{2^k}(b_{k+1})|>1$. When *k* is large this implies that

$$\begin{aligned} b_{k+1}^{\beta -1}|DE_k(f(b_{k+1}))|\approx |Df^{2^k}(b_{k+1})|>1. \end{aligned}$$

Since $|Df^{2^k}(a_{k+1})|\approx |a_{k+1}|^{\alpha -1} |DE_k(f(a_{k+1}))|$ and $f(a_{k+1})=f(b_{k+1})$ it follows that

16 $$\begin{aligned} Df^{2^k}(a_{k+1})> C_. |a_{k+1}|^{\alpha -1} b_{k+1}^{1-\beta } \text{ and } |DE_k(f(b_{k+1}))|> C_. b_{k+1}^{1-\beta }. \end{aligned}$$

Diffeomorphic branches of maps with negative Schwarzian derivative expand cross-ratios, see [46, Chapter IV]. Applying this fact to the diffeomorphism $E_k\circ f_1 :[a_{k+1},e_k] \rightarrow [b_{k+1},B_k]$ and the four points $a_{k+1},a_{k+1}^+,d_k,e_k$ (which map to $b_{k+1},b_{k+1}^+,b_k,B_k$) (where we take $a_{k+1}^+=a_{k+1}+h$ with $h>0$ close to 0 and $b_{k+1}^+$ the image of this point) we obtain the inequality

$$\begin{aligned} \dfrac{(e_k-a_{k+1}^+)(d_k-a_{k+1})}{(a_{k+1}-a_{k+1}^+)(e_k-d_k)} \le \dfrac{(B_k-b_{k+1}^+)(b_k-b_{k+1})}{(b_{k+1}-b_{k+1}^+)(B_k-b_k)} . \end{aligned}$$

Taking $h\downarrow 0$, we get

17 $$\begin{aligned} d_k&<d_k-a_{k+1}\le \dfrac{(B_k-b_{k+1})}{(B_k-b_k)}(b_k-b_{k+1})\dfrac{(e_k-d_k)}{(e_k-a_{k+1})} \dfrac{1}{Df^{2^{k}}(a_{k+1})} \nonumber \\&\le C b_{k+1}^{\beta -1} b_k. \end{aligned}$$

Here we use that the first factor in the long expression is bounded from above by Lemma 3, the second by $b_k$, the third factor by 1 and in the final factor we use the bound from (16). $\quad \square$

### Some dichotomies

**Fig. 6:**
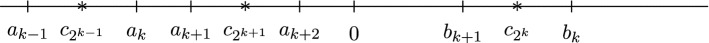
The ordering of several dynamically relevant point; here *k* is even

*For convenience of the reader we have indicated the position of the points* $a_k$, $b_k$*etc in Fig.* 6.

#### Lemma 6

There exists a constant $C > 0$ such that for large even values of *k*,

$$\begin{aligned} |A_{k+2}| > \min (C b_{k+1}, \frac{1}{2} |a_k |). \end{aligned}$$

#### Proof

Note that $E_{k+2}=E_{k+1}\circ f_1\circ E_{k+1}|T_{k+2}$ and that $E_{k+1}$ maps ${{\hat{J}}}_{k+1}\ni f(0)$ diffeomorphically onto $[{{\hat{A}}}_{k+1},{{\hat{B}}}_{k+1}]= [c_{2^{k-1}},c_{2^k}] \supset [a_k,c_{2^k}]$.

If $d_k\le c_{2^k}$ then the last interval contains $[a_k,d_k]$. Moreover, $E_k \circ f_1$ maps $[a_k,d_k]$ diffeomorphically to $[a_k,b_k]\supset [0,b_k]$ and the latter interval is mapped diffeomorphically by $f^{2^k}$ to $[a_k,c_{2^k}]$. Since $E_{k+1}=f^{2^k}\circ E_k \circ f_1|T_{k+1}$, it follows that $A_{k+2} \le a_k$ and since both numbers are negative we get $|A_{k+2}|\ge |a_k|$.

If $d_k>c_{2^k}$ then the same consideration shows that $A_{k+2}=E_{k+1}\circ f_1(c_{2^k})$. If $|A_{k+2}|>\frac{1}{2}|a_k|$ or $|A_{k+2}|>\frac{1}{2}b_{k+1}$ there is nothing to prove. So in the remainder of the proof of this lemma assume that $|A_{k+2}|\le \frac{1}{2}|a_k|$ and $|A_{k+2}|\le \frac{1}{2}b_{k+1}$. The interval $[A_{k+2},a_{k+2}]$ is well-inside the interval $[a_k,c_{2^k}]$ as $c_{2^k}>b_{k+1}>2|A_{k+2}|$ and $|a_k|\ge 2|A_{k+2}|$. Moreover, $[{{\hat{A}}}_{k+1},{{\hat{B}}}_{k+1}]=[c_{2^{k-1}},c_{2^k}]$ is the diffeomorphic range of $E_{k+1}|{{\hat{J}}}_{k+1}$, $[c_{2^{k-1}},c_{2^k}]\supset [a_k,c_{2^k}]$ and $[f(a_{k+2}),f_1(c_{2^k})]\subset {{\hat{J}}}_{k+1}$. So $[A_{k+2},a_{k+2}]=E_{k+1} [f(a_{k+2}),f_1(c_{2^k})]$ is well-inside the diffeomorphic range of $E_{k+1}|{{\hat{J}}}_{k+1}$ and so the distortion of $E_{k+1}$ restricted to $[f(a_{k+2}),f_1(c_{2^k})]$ is bounded.

It follows that the distortion of $E_{k+1}\circ f_1 |_{[a_{k+2},c_{2^k}]}$ is bounded. Since the derivative of $f^{2^{k+1}}$ at its fixed point $a_{k+2}$ is larger than one, this implies that $|D(E_{k+1}\circ f_1)(x)|>C_5$ for all $x\in [a_{k+2},c_{2^k}]$. Since $a_{k+2}<0<b_{k+1}<c_{2^k}$, $E_{k+1}$ is orientation reversing and $E_{k+1}\circ f_1(0)=c_{2^{k+1}}<0$,

$$\begin{aligned}&|A_{k+2}|=|E_{k+1}\circ f_1(c_{2^k})|>|E_{k+1}\circ f_1(b_{k+1})|>C_5 b_{k+1}. \end{aligned}$$

$$\square $$

#### Lemma 7

There exists $C > 0$ such that the following holds. Let *k* be a sufficiently large even integer. Then either

- $$|A_k | > C b_{k+1}$$ or
- $$e_k < b_{k+1}$$.

#### Proof

Suppose $e_k\ge b_{k+1}$. Then due to Lemma 4(2) and inequality (15) from Lemma 5, we know that for *k* large and even,

18 $$\begin{aligned} b_{k+1}\le e_k< d_{k-2}< C_4 b_{k-1}^{\beta -1}b_{k-2}<b_{k-2}^\beta . \end{aligned}$$

From Lemma 6 we know that either $|A_k|>Cb_{k-1}$ or $|A_k|>\frac{1}{2} |a_{k-2}|$. In the first case we have nothing to do because $b_{k+1}<b_{k-1}$. In the second case it follows from (18) that $|A_k|>\frac{1}{2} |a_{k-2}|> C b_{k-2}^\beta >C_6 b_{k+1}$. $\quad \square$

### Conditional first universal bounds

#### Lemma 8

For any $C > 0$ there exist $0< \lambda _1< \lambda _2 < 1$ such that the following holds. Let *k* be large even integer and $|A_k | > Cb_{k+1}$. Then

19 $$\begin{aligned} |Df^{2^k} |[b_{k+1},b_k ] |&> \lambda _1 \,\,\, , \end{aligned}$$

20 $$\begin{aligned} \lambda _1 b_k< b_{k+1}&< \lambda _2 b_k . \end{aligned}$$

#### Proof

Consider two cases.

Case 1: $|a_k | < \frac{1}{2} Cb_{k+1}$. Then $|b_{k+1} - a_k | < (1 + \frac{1}{2} C)b_{k+1}$. At the same time $|A_k - a_k | > \frac{1}{2} C b_{k+1}$ and we see that $|A_k - a_k | > C_7 |b_{k+1} - a_k |$ for some $C_7 > 0$ which depends only on *C*.

Case 2: $|a_k | \ge \frac{1}{2} Cb_{k+1}$. Then $|b_{k+1} -a_k | \le (1+ \frac{2}{C} )a_k$. According to Lemma 3, $|A_k | > K|a_k |$ for some universal $K > 1$, therefore $|A_k - a_k | > (K - 1)|a_k |$ and we again get $|A_k - a_k | > C_8 |b_{k+1} - a_k |$ for some $C_8 > 0$ which depends only on *C* and *K*.

From this and Lemma 3, we get that the range of the map $E_k :[f (b_{k+1}), f (b_k )] \rightarrow [a_k , b_{k+1}]$ can be diffeomorphically semi-extended to a $C_9$-scaled neighbourhood of the interval $[a_k , b_{k+1}]$, and therefore the distortion of the map $E_k |[f (b_{k+1}),f (b_k )]$ is bounded.

On the interval $[b_{k+1}, b_k ]$ the absolute value of *Df* is increasing, hence

$$\begin{aligned} |Df^{2^k} (x)| = |DE_k (f (x))| |Df (x)| > C_{10} |Df^{2^k} (b_{k+1})| \end{aligned}$$

for all $x \in [b_{k+1} , b_k ]$ and some constant $C_{10} > 0$ which depends only on *C*. Since $b_{k+1}$ is a repelling fixed point of $f^{2^k}$, we get $|Df^{2^k} (b_{k+1})| > 1$ and $|Df^{2^k}|>C_{10}$ on $[b_{k+1} ,b_k ]$. This implies the existence of $\lambda _1>0$ as in Eqs. (19) and (20).

To prove the existence of $\lambda _2<1$ in (20), note that by Lemma 3 and Koebe that $E_k$ has bounded distortion on the range $[b_k/2,b_k]$. Moreover, $f_2$ has bounded distortion on $[b_k/2,b_k]$. By contradiction assume that $b_{k+1}/b_k\approx 1$. Then there exists a point $x\in [b_{k+1},b_k]$ for which $(E_k\circ f_2)(x)\in [b_k/2,b_{k+1}]$ and $|D(E_k\circ f_2)(x)|$ is large. But since $(E_k\circ f_2)(y)\in [b_{k+1},b_k]$ for all $y\in [b_{k}/2,b_{k+1}]$, it follows that $|D(E_k\circ f_2)(y)|$ is also large for all such *y*. But this contradicts that $(E_k\circ f_2)$ maps $[b_k/2,b_{k+1}]$ into $[b_{k+1},b_k]$. Thus the existence of $\lambda _2<1$ follows. $\quad \square$

### Getting space some of the time

Now we are ready to combine the results from the previous subsection.

#### Lemma 9

There exists a constant $C > 0$ and an infinite sequence of even integers $k_1< k_2 < \dots$ such that

$$\begin{aligned} |A_{k_i} | > Cb_{k_i} , \end{aligned}$$

and therefore, the distortion of the maps $E_{k_i} |J_{k_i}$ is universally bounded.

#### Proof

It follows from Lemma 7 that either there exist infinitely many even integers $k_i$ such that $|A_{k_i} | > Cb_{k_i +1}$ or there exists an even integer $k_0$ such that $e_k < b_{k+1}$ for all even $k \ge k_0$.

In the first case we are done because of Lemmas 3 and 8, so suppose that we are in the second case. Since $0 < e_{k+1} \le e_k$, Lemma 4(5) implies $b'_{k+2}< b_{k+1}$ and $A_{k+2}= A_{k+1}$ for all even $k \ge k_0$ . Notice that $b_{k+1} < c_{2^k}=B_{k+2}$, and therefore Lemma 4(1) gives $b'_{k+2} < B_{k+2}$ . Then from Lemma 4(4) it follows that $A_{k+3} = A_{k+2}$ . So, we see that $A_k = A_{k_0 +1}$ for all $k > k_0$ and since $b_k \rightarrow 0$ we get $|A_k | > b_k$ for all *k* large enough.

The boundedness of the distortion of the maps $$E_{k_i} |J_{k_i}$$ follows from Lemma 3 and from $$|A_{k_i} | > Cb_{k_i} $$. $$\quad \square $$

### Space for some *k* gives improved space for the next *k*

#### Lemma 10

For every constant $C > 0$ there exists a constant $\tau _* > 0$ such that the following holds. Let *k* be a large even integer and $|A_k| > Cb_k$. Then

21 $$\begin{aligned} b_{k+2}&< \tau _* b_k^{2-1/\beta } , \end{aligned}$$

22 $$\begin{aligned} b_k - c_{2^k}&< \tau _* b_k^\beta \quad \,\,\, , \end{aligned}$$

23 $$\begin{aligned} d_k&< \tau _* b_k^{2\beta -1} \,\, . \end{aligned}$$

#### Proof

Due to Lemma 3 we always have some space to the right of the renormalization interval, and since we assumed that $|A_k| > Cb_k$, therefore the distortion of the map $E_k|J_k$ is bounded by a constant depending only on *C*. The map $E_{k+1}|J_{k+1}$ can be decomposed as $E_{k+1}|J_{k+1} = E_k|J_k \circ f|[b_{k+1},b_k] \circ E_k|J_{k+1}$. Due to Lemma 8 we know that $b_{k+1} > \lambda _1b_k$, and hence, the distortion of the map $f|[b_{k+1},b_k]$ is bounded. Thus, the distortion of $E_{k+1}|J_{k+1}$ is bounded as a composition of three maps of bounded distortion. Then the distortion of the map $f^{2^{k+1}} |[a_{k+1},0]$ is bounded again. Combining this with $f^{2^{k+1}}(a_{k+1}) = b_{k+1}$ and $f^{2^{k+1}}(0) = c_{2^{k+1}} \in [a_{k+1},a_{k+2}]$ we get

24 $$\begin{aligned} Df^{2^{k+1}} |[a_{k+1},0] > C_{11}b_{k+1}/|a_{k+1}|. \end{aligned}$$

This implies the following estimate on the position of $a_{k+2}$ and, therefore, of $b_{k+2}$:

25 $$\begin{aligned} \begin{array}{rllrll} |a_{k+2}| &{}< &{} \frac{|a_{k+1}|^2}{C_{11}b_{k+1}} &{}< &{} C_{12}b_k^{2\beta -1}, \\ |b_{k+2}| &{} &{} < &{} &{} C_{13}b_k^{2-1/\beta } , \end{array} \end{aligned}$$

for some universal constants $C_{12} > 0$ and $C_{13} > 0$.

Since *k* is even we know that $c_{2^k} \in [b_{k+1},b_k]$ and $c_{2^{k+1}} \in [a_{k+1},a_{k+2}]$ and so in particular $f^{2^k}[c_{2^k},b_k]\subset [a_k,0]$. Due to Lemma 8 the derivative of $f^{2^k}|[b_{k+1},b_k]$ is bounded away from zero, hence

26 $$\begin{aligned} |b_k - c_{2^k}|< \lambda _1^{-1} |a_k|<C_{14} b_k^\beta \ll b_k \end{aligned}$$

for some universal constant $C_{14}$. Combining this with Eq. (24), and since $f^{2^k}[0,d_k]=[c_{2^k},b_k]$, this gives us a much better estimate for $d_k$ (compared to inequality (5)):

27 $$\begin{aligned} d_k< C_{11}^{-1}|b_k - c_{2^k}| \cdot |a_{k+1}| / b_{k+1}< C_{15}b_k^\beta |a_{k+1}| / b_{k+1} < C_{15} b_k ^{2\beta -1} \end{aligned}$$

for some $$C_{15} > 0$$. $$\quad \square $$

#### Lemma 11

For every constant $C_0 > 0$ there exists a constant $\tau _* > 0$ such that the following holds. Let *k* be a large even integer, *C* be a constant greater that $C_0$, and $|A_k| > Cb_k$, $B_k > (1 + C)b_k$. Then

$$\begin{aligned}|A_{k+2}| > \tau _* \min (C, b_k^{1-\beta })b_k.\end{aligned}$$

#### Proof

Set

28 $$\begin{aligned} {{\tilde{A}}}_k= & {} -\frac{1}{2}Cb_k \nonumber \\ {\tilde{B}}_k= & {} (1+\frac{1}{2}C) b_k. \end{aligned}$$

Let ${{\tilde{e}}}_k, {{\tilde{b}}}_k$ be points such that $E_k \circ f_1({{\tilde{e}}}_k) = {{\tilde{B}}}_k$ and $E_k \circ f_2({{\tilde{b}}}_k) = {{\tilde{A}}}_k$. Arguing as before we see that the distortions of maps $E_k \circ f_1|[a_k,{{\tilde{e}}}_k]$ and $E_k \circ f_2|[b_{k+1},\tilde{b}_k]$ are bounded by some constant depending on $C_0$. Therefore, for all $x\in [a_k,{{\tilde{e}}}_k]$,

29 $$\begin{aligned} D(E_k \circ f_1)(x)> & {} C \dfrac{b_k-a_k}{ d_k - a_k} \nonumber \\> & {} C_{17} b_k^{1-\beta }. \end{aligned}$$

In the same way we get the estimate on the derivative of the other branch:

$$\begin{aligned} D(E_k \circ f_2)(x) > C_{18} \end{aligned}$$

for all $x\in [b_{k+1},{{\tilde{b}}}_k]$. Now consider the following cases.

**Case 1.a.** Assume that ${{\tilde{e}}}_k < b_{k+1}$ and ${{\tilde{B}}}_k > {{\tilde{b}}}_k$. Then, arguing as in Lemma 4(4,5) we obtain that $|A_{k+2}| > |{{\tilde{A}}}_k|$ and we are done in this case.

**Case 1.b.** Now suppose ${{\tilde{e}}}_k < b_{k+1}$ and $\tilde{B}_k \le {{\tilde{b}}}_k$. Then

30 $$\begin{aligned} |E_{k+1} \circ f_1([d_k, {{\tilde{e}}}_k])|> & {} C_{18}|{{\tilde{B}}}_k - b_k|\nonumber \\= & {} \dfrac{1}{2}C_{18}Cb_k. \end{aligned}$$

Using an argument similar to prove Lemma 2(4) we get $|A_{k+2}| > \frac{1}{2} C_{18}C b_k$ and this case is also done.

**Case 2:** ${{\tilde{e}}}_k > b_{k+1}$. From the derivative estimate we know

31 $$\begin{aligned} E_k \circ f_1([d_k, b_{k+1}])> & {} C_{17}b_k^{1-\beta }|b_{k+1} - d_k| \nonumber \\> & {} C_{19} b_k^{2-\beta } . \end{aligned}$$

Here we used inequalities (20) and (23).

We finish by considering two subcases as in Case 1. If $E_k \circ f_1(b_{k+1}) > {{\tilde{b}}}_k$, then as before $|A_{k+2}| > |\tilde{A}_k|$. Otherwise,

$$\begin{aligned}&|A_{k+2}| > C_{18}C_{19}b_k^{2-\beta }. \qquad \qquad \end{aligned}$$

$$\square $$

### The proof of the first part of Theorem 3: getting huge space all the time

The following lemma completes the proof of the first part of the ‘Big Bounds’ Theorem 3. The actual bounds for the space that are claimed in that theorem will be only obtained in the improved bounds from Lemma 13.

#### Lemma 12

(Koebe Space for the semi-extension) There exists ${{\hat{\lambda }}}>0$ so that as *k* even and $k\rightarrow \infty$,

32 $$\begin{aligned} \dfrac{|b_{k+2}-a_{k+2}|}{|a_{k+2} -A_{k+2}|} = O(b_{k}^{1-1/\beta }), \dfrac{|b_{k+2}-a_{k+2}|}{|B_{k+2} -b_{k+2}|} = O(b_{k}^{1-1/\beta }) \end{aligned}$$

and

33 $$\begin{aligned} \dfrac{|b_{k+1}-a_{k+1}|}{|a_{k+1} -A_{k+1}|} = O(b_{k-2}^{1-1/\beta }) , \dfrac{|b_{k+1}-a_{k+1}|}{ |B_{k+1} - b_{k+1}|} \ge {{\hat{\lambda }}} . \end{aligned}$$

In particular, the range of the map $E_k|J_k$ can be monotonically semi-extended to a $\tau _k$ scaled neighbourhood of $[a_k,b_k]$ where $\tau _k \approx O(b_{k-2}^{1-1/\beta })$ for *k* even and $\tau _k \approx 1$ for *k* odd.

Moreover, $O(b_{k}^{1-1/\beta })$ converges super-exponentially to zero: $\log (b_{k})$ converges exponentially to zero.

#### Proof

This lemma is a consequence of the previous two lemmas. Let *k* be a large (even) integer from the sequence given by Lemma 9. Then, from Lemmas 10 and 11 it follows that

34 $$\begin{aligned} |A_{k+2}|> & {} C_{20}b_k^{\frac{1}{\beta }-1} b_{k+2}, \nonumber \\ |B_{k+2}|> & {} C_{20}b_k^{\frac{1}{\beta }-1} b_{k+2}, \end{aligned}$$

for some universal constant $C_{20} > 0$. Since $\beta > 1$ we see that if k is large enough, we get huge improvement on the relative size of extension interval $[A_{k+2},B_{k+2}]$ compared to the renormalization interval $[a_{k+2},b_{k+2}]$. From this point the argument can be applied inductively and (32) follows.

Lemma 8 gives $|a_{k+1}-b_{k+1}|\approx |a_{k}-b_{k}|$. By the proof of Lemma 4(4) either $A_{k+1}=A_k$ (if $b_k'<B_k$) or $A_{k+1}=E_k\circ f_2(B_k)$ (if $B_k\le b_k'$). In the former case we use (32) and get $\dfrac{|b_{k+1}-a_{k+1}|}{|a_{k+1} -A_{k+1}|}\approx \dfrac{|b_{k}-a_{k}|}{|a_{k} -A_{k}|} =O(b_{k-2}^{1-1/\beta })$. So let us check what happens when $B_k\le b_k'$. Using (34) we obtain (*) $\dfrac{|f(0)-f_2(b_k)|}{|f(0)-f(B_k)|}\approx b_{k}^\beta / B_{k}^\beta = O(b_{k-2}^{\beta -1})$. On the other hand, the expression in (32) and Koebe imply $\dfrac{|x-f_2(b_{k})|}{|f_2(b_{k})-f_2(b_{k}')|} = O(b_{k-2}^{1-1/\beta })$ where *x* is so that $E_{k}(x)=b_{k}$, see Fig. 5. Since $c_{2^k}\sim b_k$ we have $|x-f(a_{k}|\approx |f(a_k)-f(0)|$ this implies (**) $\dfrac{|f(0)-f_2(b_{k})|}{|f_2(0)-f_2(b_{k}')|} = O(b_{k-2}^{1-1/\beta })$. Since $b_{k-2}^{1-1/\beta }>> b_{k-2}^{\beta -1}$ and comparing (*) and (**) we can conclude that either $B_k>b_k'$ or (by Koebe) $E_k\circ f_2(B_k)|\ge (1/2)|A_k|$. In either case (33) holds.

Since $B_{k+1}=c_{2^k} \sim b_k$, we have by (20) that there exist universal constants $0<\lambda _1'<\lambda _2'<1$ so that $\dfrac{|b_{k+1}-a_{k+1}|}{ |B_{k+1} - b_{k+1}|} \sim \dfrac{|b_{k+1}|}{ |b_k - b_{k+1}|}\in (\lambda _1',\lambda _2')$. Which proves the second expression in (33) and that this expression cannot be improved.

The final statement follows from inequality (21). $\quad \square$

## Scaling Laws, Renormalization Limits and Universality

**A first error bound for the map** $$f^{2^k}$$**on** $$[a_k,b_k]$$**when** *k* **is even.** Let *k* be even and $$x_k$$ be so that $$E_k(x_k)=b_k$$, see Fig. 5. Then $$E_k:[f(a_k),x_k]\rightarrow [a_k,b_k]$$ is the first entry map and $$\tau _k$$ be the Koebe space of $$E_k|[f(a_k),x_k]$$. Let $$L_k$$ be the affine map which agrees with $$E_k$$ on the boundary points of $$ [f(a_k),f(0)]$$. By the Corollary of Koebe, Lemma 2, we obtain for all $$x\in [f(a_k),f(0)]$$

35 $$\begin{aligned} E_k(x) =L_kx+O(b_k/\tau _k) \text{ and } DE_k(x)=DL_k(1+O(1/\tau _k)). \end{aligned}$$

By Lemma 12$\tau _k\approx b_{k-2}^{1/\beta -1}\rightarrow \infty$. In particular it follows that $O(b_k/\tau _k)=o(b_k)$. Obviously $DL_k\approx b_k/|a_k|\approx b_k^{1-\beta }$. Hence

36 $$\begin{aligned} E_k(x)= L_kx+o(b_k) \text{ and } DE_k(x) \sim DL_k, \end{aligned}$$

for all $x\in [f(a_k),f(0)]$. Later on, we will improve the error bound in this expression. Hence

37 $$\begin{aligned} f^{2^k} (x) = {\left\{ \begin{array}{ll} c_{2^k} - s_k |x| +o(b_k) &{} \text{ when } x\in [a_k,0],\\ c_{2^k} - t_k x^\beta +o(b_k) &{} \text{ when } x\in [0,b_k], \end{array}\right. } \end{aligned}$$

where $s_k>0$ is so that $c_{2^k}-s_k|a_k| + o(|b_k|)=-|a_k|$ and $t_k>0$ is so that $c_{2^k}-t_k b_k^{\beta }+o(|b_k|)=-|a_k|$. By (22) we have $c_{2^k}=b_k+O(b_k^\beta )\sim b_k$ and since $a_k\sim -K_0 b_k^{\beta }$, this implies

38 $$\begin{aligned} s_k\sim \dfrac{b_k^{1-\beta }}{K_0} \text{ and } t_k\sim b_k^{1-\beta }. \end{aligned}$$

Equation (36) also gives

39 $$\begin{aligned} Df^{2^k} (x) \sim {\left\{ \begin{array}{ll} s_k&{} \text{ when } x\in [a_k,0),\\ -t_k \beta x^{\beta -1} &{} \text{ when } x\in (0,b_k]. \end{array}\right. } \end{aligned}$$

For simplicity we will write

$$\begin{aligned} f_{l,k}:=f^{2^k}|[a_k,0] \text{ and } f_{r,k}:=f^{2^k}|[0,b_k]. \end{aligned}$$

To avoid an overload of notation we usually write

$$\begin{aligned} f_l=f_{l,k} \text{ and } f_r = f_{r,k} \end{aligned}$$

if it clear from the context which *k* is used.

**The scaling law from** $b_k$**to** $b_{k+1}$**when** *k* **is even.** Write $b_{k+1}=\lambda _{k} b_k$. Then (37) implies

40 $$\begin{aligned} c_{2^k}-t_k \lambda _{k}^\beta b_k^\beta +o(b_k) =f^{2^k}(b_{k+1}) = b_{k+1}=\lambda _k b_k . \end{aligned}$$

By (22)

$$\begin{aligned} c_{2^k}=b_k+O(b_k^\beta ) \end{aligned}$$

and combining this with (38) and (40) implies

$$\begin{aligned} 1-\lambda _{k}^\beta +o(1)=\lambda _k . \end{aligned}$$

So taking $\lambda \in (0,1)$ be the root of $1-\lambda ^\beta =\lambda$ this gives $\lambda _k=\lambda +o(1)$ and

$$\begin{aligned} b_{k+1}=\lambda b_k + o(b_k). \end{aligned}$$

Later on we will improve on this statement, see (58).

**The approximate scaling law from** $b_k$**to** $b_{k+2}$**when** *k* **is even.** Fix some $\delta >0$ and let $C_k$ be so that $c_{2^{k+1}}= - C_k b_k^{\delta }$. Below we will determine $\delta$ and $C_k$. Note that

$$\begin{aligned} a_{k+1}<c_{2^{k+1}}<0<c_{2^{k+2}}<b_{k+2}<b_{k+1}<c_{3\cdot 2^k}<c_{2^k}<b_k. \end{aligned}$$

Then using (38) and (39)

41 $$\begin{aligned} c_{2^k}-c_{3\cdot 2^{k}}=f^{2^k}(0)-f^{2^k}(c_{2^{k+1}})=f_l(0)- f_l(c_{2^{k+1}})\sim \dfrac{C_k}{K_0} b_k^{\delta } b_k^{1-\beta }. \end{aligned}$$

Since $f_r$ has bounded distortion and bounded derivative on $[b_{k+1},b_k]$ this implies

42 $$\begin{aligned} c_{2^{k+2}}-c_{2^{k+1}} = f_r\circ f_l (c_{2^{k+1}}) - f_r(c_{2^k}) = f_r(c_{3\cdot 2^k})-f_r(c_{2^k}) \approx C_k b_k^{\delta } b_k^{1-\beta } . \end{aligned}$$

In fact,

43 $$\begin{aligned} |c_{2^k}-c_{3\cdot 2^k}| \approx |c_{2^{k+2}}-c_{2^{k+1}}|< |b_{k+2}-a_{k+1}| <o(b_k) \end{aligned}$$

where $\approx$ follows from the fact that $Df_r$ is bounded from above and below on $[b_{k+1},b_k]$, where the first < follows from the ordering of the points and where $<o(b_k)$ follows from Eq. (21) and $|a_{k+1}|\approx b_{k+1}^\beta$. Combining this with $c_{2^k}\sim b_k$, Eqs. (39) and (38) give $f_r'(b_k)\sim -\beta$ and $f_r'(x)\sim -\beta$ for all $x\in [c_{3\cdot 2^{k}},c_{2^k}]$. Hence (42) in fact improves to

44 $$\begin{aligned} c_{2^{k+2}}-c_{2^{k+1}} \sim \dfrac{\beta C_k}{K_0} b_k^{\delta } b_k^{1-\beta } . \end{aligned}$$

Since $|c_{2^{k+1}}|=C_kb_k^\delta<< \dfrac{\beta C_k}{K_0} b_k^{\delta } b_k^{1-\beta }$ and using that $b_{k+2}\sim c_{2^{k+2}}$, Eq. (44) gives

45 $$\begin{aligned} b_{k+2}\sim c_{2^{k+2}}\sim \dfrac{\beta C_k}{K_0} b_k^{\delta } b_k^{1-\beta } \text{ and } a_{k+2} \sim -K_0 [\dfrac{\beta C_k}{K_0} b_k^{\delta } b_k^{1-\beta }]^\beta . \end{aligned}$$

Next note that $f^{2^{k+1}}(a_{k+2})=f_r\circ f_l(a_{k+2})$. Using that $f_l|[a_k,0]$ has derivative everywhere $\sim \frac{1}{K_0} b_k^{1-\beta }$ and Eq. (21) we have that $|a_{k+2}|\le K_0 |b_{k+2}|^\beta < C |b_k|^{2\beta -1}$ and therefore Eq. (45) implies

$$\begin{aligned} f_l(a_{k+2})-f_l(0) \le C b_k^{2\beta -1} b_k ^{1-\beta }= C b_k^\beta . \end{aligned}$$

Therefore $f_l(a_{k+2})\sim b_k$ and so Eq. (39) implies

46 $$\begin{aligned} f_r'(x)\sim -\beta \text{ for } \text{ all } x\in [f_l(a_{k+2}),b_k]. \end{aligned}$$

Since, by (45),

$$\begin{aligned} f_l(a_{k+2})-f_l(0) \sim \dfrac{b_k^{1-\beta }}{K_0} K_0 [\dfrac{\beta C_k}{K_0} b_k^{\delta } b_k^{1-\beta } ]^\beta = \left[ \dfrac{\beta C_k}{K_0}\right] ^{\beta } b_k ^{\beta \delta +1-\beta ^2}. \end{aligned}$$

Hence (46) implies

47 $$\begin{aligned} f^{2^{k+1}}(a_{k+2})-c_{2^{k+1}}=f_r\circ f_l(a_{k+2})-f_r(f_l(0))\sim \beta \left[ \dfrac{\beta C_k}{K_0}\right] ^{\beta } b_k ^{\beta \delta +1-\beta ^2}. \end{aligned}$$

By (45), $f^{2^{k+1}}(a_{k+2})=a_{k+2}\approx -C_k^\beta [b_k^{\delta } b_k^{1-\beta }]^\beta = -C_k^\beta b_k^{\beta \delta +\beta -\beta ^2}$ is orders smaller than the right hand side of (47), and thus it follows that

48 $$\begin{aligned} c_{2^{k+1}} \sim - \beta \left[ \dfrac{\beta C_k}{K_0}\right] ^{\beta } b_k ^{\beta \delta +1-\beta ^2}. \end{aligned}$$

Using $c_{2^{k+1}}= - C_k b_k^{\delta }$ we obtain as a natural choice

49 $$\begin{aligned} \delta =\beta \delta +1-\beta ^2 \text{ which } \text{ gives } \delta =\beta +1 \end{aligned}$$

and

50 $$\begin{aligned} C_k\sim \beta \left[ \dfrac{\beta C_k}{K_0}\right] ^{\beta } \text{ and } \text{ therefore } C_k\sim \left[ \dfrac{K_0^\beta }{\beta ^{\beta +1}} \right] ^{1/(\beta -1)} . \end{aligned}$$

Hence from (45), $b_{k+2}\sim c_{2^{k+2}}$ and $c_{2^{k+1}}= - C_k b_k^{\delta }$ we obtain

51 $$\begin{aligned} b_{k+2} \sim \dfrac{\beta }{K_0} \left[ \dfrac{K_0^\beta }{\beta ^{\beta +1}} \right] ^{1/(\beta -1)} b_k^2 = \beta ^{-2/(\beta -1)}K_0^{1/(\beta -1)} b_k^2 \end{aligned}$$

and

52 $$\begin{aligned} c_{2^{k+1}}\sim -\left[ \dfrac{K_0^\beta }{\beta ^{\beta +1}} \right] ^{1/(\beta -1)} b_k^{\beta +1}. \end{aligned}$$

Since $b_{k+1}\sim \lambda b_k$ this gives

53 $$\begin{aligned} b_{k+2} \sim \beta ^{\frac{-2}{\beta -1}} K_0^{\frac{1}{\beta -1}} \lambda ^{-2} b_{k+1}^2 \end{aligned}$$

and

54 $$\begin{aligned} c_{2^{k+1}}\sim -\beta ^{-\frac{\beta +1}{\beta -1}} K_0^{\frac{\beta }{\beta -1}} \lambda ^{-\beta -1} b_{k+1}^{\beta +1}. \end{aligned}$$

**The usual Koebe space does not hold and the proof of Theorem** 2 Let $T\ni f(0)$ be the maximal interval on which $f^{2^k -1}|T$ is diffeomorphic. Then by Lemma 4 we have that $f^{2^k-1}=[{{\hat{A}}}_k,{{\hat{B}}}_k]\supset [a_k,b_k]$ where

$$\begin{aligned} {{\hat{A}}}_k= & {} c_{2^{k-1}}, {{\hat{B}}}_k=c_{2^{k-2}} \text{ when } k \text{ is } \text{ even }\\ {{\hat{A}}}_k= & {} c_{2^{k-2}}, {{\hat{B}}}_k=c_{2^{k-1}} \text{ when } k \text{ is } \text{ odd }. \end{aligned}$$

When *k* is even then

$$\begin{aligned} {{\hat{A}}}_k=c_{2^{k-1}}\approx b_{k-1}^{\beta +1} \approx b_k^{(\beta +1)/2}=o(b_k) \end{aligned}$$

and when *k* is odd then

$$\begin{aligned} {{\hat{A}}}_k=c_{2^{k-2}}\approx b_{k-2}^{\beta +1} \approx b_k^{(\beta +1)/2}=o(b_k). \end{aligned}$$

So in either case there exists no $\tau >0$ so that $[{{\hat{A}}}_k,{{\hat{B}}}_k]$ is a $\tau$-scaled neighbourhood of $[a_k,b_k]$ for *k* large. In other words, there is no Koebe space (on the left) for the diffeomorphic extension of the first entry map into $[a_k,b_k]$.

**Improved Koebe Space for the semi-extension and the proof of Theorem 3 (Big Bounds).** We can now prove Theorem 3 and an improved version of Lemma 12:

### Lemma 13

(Improved Koebe Space) The range of the map $E_k|J_k$ can be monotonically semi-extended to a $\tau _k$ scaled neighbourhood of $[a_k,b_k]$ where $\tau _k \approx b_{k-2}/b_{k} \approx b_k^{-1/2}$ when *k* is even and $\tau _k \approx 1$ for *k* odd.

### Proof

The map $E_k|J_k$ can be monotonically semi-extended onto $[A_k,B_k]$. As we saw in Lemmas 11 and 12 we have $|A_k|\ge b_{k-2}$ for *k* even. By Lemma 4 and the previous bounds, we have for *k* even $B_k=c_{2^{k-2}}\approx b_{k-2}$. It follows from this and (51) that $\tau _k\approx b_{k-2}/b_k \approx b_k^{-1/2}$. Note that for *k* odd, $B_k=b_{k-1}$ and so $\tau _k = b_k/B_k = b_k/b_{k-1}\rightarrow \lambda$ as $k\rightarrow \infty$ and *k* odd. $\quad \square$

**Proof of Theorems** 5**and** 6**(Renormalization limits of** $R^k$)**:** Given the previous lemma, we obtain that the Koebe space is of the order $\tau _k\approx b_k^{-\frac{1}{2}}$. It follows that $O(b_k/\tau _k)=O(b_k^{\frac{3}{2}})$ and so (35) gives

55 $$\begin{aligned} f^{2^k} (x) = {\left\{ \begin{array}{ll} c_{2^k} - s_k |x| +O(b_k^{\frac{3}{2}}) &{} \text{ when } x\in [a_k,0]\\ c_{2^k} - t_k x^\beta +O(b_k^{\frac{3}{2}}) &{} \text{ when } x\in [0,b_k] \end{array}\right. } \end{aligned}$$

with

56 $$\begin{aligned} s_k\sim \dfrac{b_k^{1-\beta }}{K_0} \text{ and } t_k\sim b_k^{1-\beta }. \end{aligned}$$

The proof of Theorem 6 follows the above and an explicit calculation. For example,

$$\begin{aligned} \lim _{k\rightarrow \infty } (R^{2k+1} f ) \circ m_{2k+1} \end{aligned}$$

is composition of the asymptotically linear left branch of $R^{2k}f$ and of the part of the right branch of $R^{2k}f$ corresponding to $[b_{k+1},c_{2^k}]$ where $c_{2^k}\sim b_k$.

**Improved scaling law from** $b_k$**to** $b_{k+1}$**when** *k* **is even.** Arguing as in (40) and below we have

57 $$\begin{aligned} c_{2^k}-t_k \lambda _{k}^\beta b_k^\beta = \lambda _k b_k + O(b_k^{\frac{3}{2}}) \end{aligned}$$

and therefore

$$\begin{aligned} b_k- \lambda _{k}^\beta b_k + O(b_k^\beta ) = \lambda _k b_k + O(b_k^{\frac{3}{2}}). \end{aligned}$$

This means

$$\begin{aligned} b_k- \lambda _{k}^\beta b_k = \lambda _k b_k + O(b_k^{\frac{3}{2}}) + O(b_k^{\beta }) \end{aligned}$$

and so

58 $$\begin{aligned} \lambda _k = \lambda + O(b_k^{\frac{1}{2}})+O(b_k^{\beta -1}) \end{aligned}$$

where as before $\lambda \in (0,1)$ is the root of $1-\lambda ^\beta =\lambda$. In the same way, we obtain that the $\sim$ expressions in this Sect. 9 are in fact equalities with a multiplicative error of the form $1+O(b_k^\epsilon )$ for some $\epsilon >0$.

One can similarly also obtain exponential convergence for the constants in the scaling for $b_{k+1}$ to $b_{k+2}$.

**The growth rate of** $\log b_k$**and the completion of the proof of Theorem** 4. Let $\mu _{k}=\log (1/ b_{2k})$. As we saw $\mu _{k}\rightarrow \infty$. Let us give a sharper estimate here. According to (51) $\mu _{k+1}=2\mu _{k} + D_{k}$ for all $k\ge 0$ where

59 $$\begin{aligned} D_{k}\sim D :=\log ( \beta ^{\frac{2}{\beta -1}} K_0^{\frac{-1}{\beta -1}}) . \end{aligned}$$

It follows that $\mu _{k}/2^k=(\mu _0 + D_{k-1}/2^{k} + \dots + D_0/2)$ and therefore there exists $\Theta >0$ so that $\dfrac{\mu _{k}}{2^k}\rightarrow \Theta$. Moreover,

$$\begin{aligned} \Theta - \mu _{k}/2^k = \sum _{i\ge k} D_{i}/2^{i+1}= \sum _{i\ge k} D/2^{i+1} + \sum _{i\ge k} (D_{i}-D) /2^{i+1}=D /2^{k} +o(1)/2^k. \end{aligned}$$

Hence

60 $$\begin{aligned} \log (1/b_{2k+1}) \sim \log (1/b_{2k}) = \mu _k = 2^k \Theta - D + o(1) \end{aligned}$$

and so using (59)

61 $$\begin{aligned} 1/b_{2k} = \beta ^{-\frac{2}{\beta -1}} K_0^{\frac{1}{\beta -1}} \exp (2^k \Theta + o(1)). \end{aligned}$$

**Necessary and sufficient invariants for** $h:\{c_{2^k}\}_{k\ge 0} \rightarrow \{{{\tilde{c}}}_{2^k}\}_{k\ge 0}$**to be Lipschitz.** Assume that $h:\{c_{2^k}\}_{k\ge 0} \rightarrow \{\tilde{c}_{2^k}\}_{k\ge 0}$ is a conjugacy between *f* and ${{\tilde{f}}}$ and is Lipschitz at 0. This implies

62 $$\begin{aligned} {{\tilde{c}}}_{2^{2k}} \approx c_{2^{2k}}, {{\tilde{c}}}_{2^{2k+1}} \approx c_{2^{2k+1}}. \end{aligned}$$

Since $b_{2k+1} \sim \lambda b_{2k}$ , $c_{2^{2k}} \sim b_{2k}$ where $\lambda \in (0,1)$ is the root of the equation $\lambda ^\beta +\lambda =1$, (62) implies

63 $$\begin{aligned} {{\tilde{b}}}_{2k}\approx b_{2k} \text{ and } {{\tilde{\lambda }}}^{-1} {{\tilde{b}}}_{2k+1}\approx \lambda ^{-1} b_{2k+1}. \end{aligned}$$

By Theorem 4 and (62) we also have

64 $$\begin{aligned} -{{\tilde{\beta }}}^{-\frac{{{\tilde{\beta }}}+1}{{{\tilde{\beta }}}-1}} \tilde{K}_0^{\frac{{{\tilde{\beta }}}}{{{\tilde{\beta }}}-1}} {{\tilde{\lambda }}}^{-\tilde{\beta }-1} {\tilde{b}}_{2k+1}^{{{\tilde{\beta }}}+1} \sim \tilde{c}_{2^{2k+1}} \approx c_{2^{2k+1}} \approx - \beta ^{-\frac{ \beta +1}{ \beta -1}} K_0^{\frac{ \beta }{\beta -1}} \lambda ^{- \beta -1} b_{2k+1}^{ \beta +1} . \end{aligned}$$

This, the 2nd expression in (63) and $b_{2k+1}\rightarrow 0$ imply that

65 $$\begin{aligned} \beta = {{\tilde{\beta }}} \text{ and } \text{ therefore } \lambda ={{\tilde{\lambda }}} . \end{aligned}$$

Finally (61) and (62) imply that

66 $$\begin{aligned} 1\approx {{\tilde{c}}}_{2^k} / c_{2^k} \sim {{\tilde{b}}}_{2k} / b_{2k} = \left[ \dfrac{K_0}{{{\tilde{K}}}_0}\right] ^{\frac{-1}{\beta -1}} \exp (2^k (\Theta - {{\tilde{\Theta }}}) + o(1) ). \end{aligned}$$

Hence

67 $$\begin{aligned} \Theta = {{\tilde{\Theta }}} . \end{aligned}$$

Thus we have shown that the existence of a Lipschitz conjugacy implies

68 $$\begin{aligned} \beta ={{\tilde{\beta }}} \text{ and } \Theta ={{\tilde{\Theta }}} . \end{aligned}$$

**Necessary and sufficient invariants for** $h:\{c_{2^k}\}_{k\ge 0}\rightarrow \{{{\tilde{c}}}_{2^k}\}_{k\ge 0}$**to be differentiable at** 0. By the previous paragraph, (68) are necessary conditions for *h* to be differentiable at 0. Let us show that these conditions are also sufficient. So assume that (68) holds. This and (61) imply

69 $$\begin{aligned} \dfrac{{{\tilde{c}}}_{2^{2k}}}{c_{2^{2k}}} \sim \dfrac{\tilde{b}_{2k}}{b_{2k}} \sim \dfrac{\beta ^{\frac{-2}{\beta -1}} K_0^{\frac{1}{\beta -1}}}{{{\tilde{\beta }}}^{\frac{-2}{{{\tilde{\beta }}}-1}} {{\tilde{K}}}_0^{\frac{1}{{{\tilde{\beta }}}-1}}} \exp (2^k (\Theta -\tilde{\Theta }) + o(1)) \sim \left( \dfrac{K_0}{\tilde{K}_0}\right) ^{\frac{1}{\beta -1}}:=\rho . \end{aligned}$$

By Theorem 4, ${{\tilde{\beta }}}=\beta$, $\tilde{\lambda }=\lambda$ and $b_{2k+1}\sim \lambda b_{2k}$, $\tilde{b}_{2k+1}\sim {{\tilde{\lambda }}} b_{2k}$ and the previous expression (and $\rho :=[K_0/{{\tilde{K}}}_0]^{\frac{1}{\beta -1}}$) we get

70 $$\begin{aligned} \dfrac{{\tilde{c}}_{2^{2k+1}}}{c_{2^{2k+1}} }&\sim \dfrac{ -\tilde{\beta }^{-\frac{{{\tilde{\beta }}}+1}{{{\tilde{\beta }}}-1}} \tilde{K}_0^{\frac{{{\tilde{\beta }}}}{{{\tilde{\beta }}}-1}} {{\tilde{\lambda }}}^{-\tilde{\beta }-1} {\tilde{b}}_{2k+1}^{{{\tilde{\beta }}}+1} }{- \beta ^{-\frac{ \beta +1}{ \beta -1}} K_0^{\frac{ \beta }{\beta -1}} \lambda ^{- \beta -1} b_{2k+1}^{ \beta +1}} =\left[ \dfrac{{{\tilde{K}}}_0}{K_0}\right] ^{\frac{\beta }{\beta -1}} \left[ \dfrac{{{\tilde{b}}}_{2k+1}}{b_{2k+1}}\right] ^{\beta +1}\sim \nonumber \\&\sim \left[ \dfrac{{{\tilde{K}}}_0}{K_0}\right] ^{\frac{\beta }{\beta -1}} \left[ \dfrac{{{\tilde{b}}}_{2k}}{b_{2k}}\right] ^{\beta +1} \sim \left[ \dfrac{{{\tilde{K}}}_0}{K_0}\right] ^{\frac{\beta }{\beta -1}} \rho ^{\beta +1} = \rho ^{-\beta } \rho ^{\beta +1} = \rho . \end{aligned}$$

**Another ratio.** Even though we shall not use this, let us calculate another ratio. Writing as before $c_{2^{2k+1}}= - C_{2k} b_{2k}^{\delta }$ we have according to (49) and (50) we have $\delta =\beta +1$ and $C_{2k}\sim \left[ \dfrac{K_0^\beta }{\beta ^{\beta +1}} \right] ^{1/(\beta -1)}$.

Hence, using (41), we obtain

71 $$\begin{aligned} c_{2^{2k}}-c_{3\cdot 2^{2k}}\sim \dfrac{C_{2k}}{K_0} b_{2k}^2 \sim \dfrac{K_0^{1/(\beta -1)}}{\beta ^{(\beta +1)/(\beta -1)}} b^2_{2k}. \end{aligned}$$

So assuming that (68) holds we have using (69)

$$\begin{aligned} \dfrac{{{\tilde{c}}}_{2^{2k}}-{{\tilde{c}}}_{3\cdot 2^{2k}}}{c_{2^{2k}}-c_{3\cdot 2^{2k}}}\sim \dfrac{\tilde{K}_0^{1/(\beta -1)}}{K_0^{1/(\beta -1)}} \dfrac{\tilde{b}_{2k}^2}{b_{2k}^2} \sim \dfrac{\tilde{K}_0^{1/(\beta -1)}}{K_0^{1/(\beta -1)}} \rho ^2 = \rho . \end{aligned}$$

**The invariants** (68) **are sufficient for the conjugacy** $h:\Lambda \rightarrow {{\tilde{\Lambda }}}$**to be differentiable at** 0, **where** $\Lambda$**is the attracting Cantor set** $\overline{\cup _{n\ge 0} f^n(0)}$. Regardless whether or not (68) holds, there exists a topological conjugacy $h:\Lambda \rightarrow {{\tilde{\Lambda }}}$ between *f* and ${{\tilde{f}}}$; in fact, in the next section we will show that $f,\tilde{f}$ do not have wandering intervals, and then we will also know that there exists a topological conjugacy *h* on the entire space. Let us show now that the conjugacy $h:\Lambda \rightarrow {{\tilde{\Lambda }}}$ is necessarily differentiable on $\Lambda$ when (68) is satisfied.

**Fig. 7:**
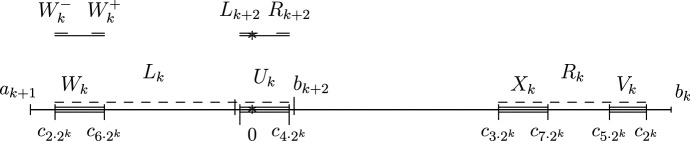
These four intervals contain the postcritical set in $[a_k,b_k]$. We will pull back the analogue of the dashed intervals for level $k+2$ inside $W_k$

To do this, note that when *k* is even that $\Lambda \cap [a_k,b_k]$ is contained in the union of following intervals $U_k,V_k,W_k,X_k$ where $U_k=[x_k,c_{4\cdot 2^{k}}]$ where $x_k<0$ is chosen so that $f(x_k)=f(c_{4\cdot 2^{k}})$ and let $U_k^-=[x_k,0]$, $U_k^+=[0,c_{4\cdot 2^{k}}]$, $V_k=f_l(U_k^-)$, $W_k=f_r(V_k)$ and $X_k=f_l(W_k)$. For simplicity also define $R_k:=[X_k,V_k]$, $L_k=[W_k,U_k]$ and $(U_k,X_k):=[c_{4\cdot 2^k},c_{3\cdot 2^k}]$. See Fig. 7 for the position of these intervals.

### Lemma 14

72 $$\begin{aligned} \liminf \dfrac{|W_k|}{|L_k|} >0. \end{aligned}$$

and

73 $$\begin{aligned} \dfrac{|R_k|}{|(U_k,X_k)|} \rightarrow 0 \text{ and } \dfrac{|L_k|}{|(U_k,X_k)|}\rightarrow 0 \text{ as } k\rightarrow \infty . \end{aligned}$$

### Proof

Note that $$|U_k^-|=|x_k|\approx |c_{4\cdot 2^k}|^\beta \sim b_{k+2}^\beta \approx b_{k}^{2\beta }$$,

$$\begin{aligned} |V_k|=|c_{2^k} - c_{5\cdot 2^k} | = |f_l(U_k^-)| \approx s_k |U_k^-| \approx b_k^{1-\beta } b_k^{2\beta } =b_k^{1+\beta } \end{aligned}$$

and by (46),

$$\begin{aligned} |W_k|=|f_r(V_k)| \approx \beta b_k^{1+\beta } \approx |c_{2^{k+1}}-0| \end{aligned}$$

where in the last $\approx$ we used (54). This implies that the size of $W_k$ is comparable to its distance to 0; in other words for any two points $u_k,v_k\in W_k$ we merely have $u_k\approx v_k$, showing (72). To prove (73), note that

$$\begin{aligned} |U_k| \sim |U_k^+| = |c_{2^{k+2}}|\sim b_{k+2} \approx b_k^2 \end{aligned}$$

and therefore

$$\begin{aligned} |L_k|=|[W_k,U_k]| \approx b_k^{1+\beta } + b_k^2 \approx b_k^2. \end{aligned}$$

Similarly, by (41) and $\delta =1+\beta$ we have

74 $$\begin{aligned} |R_k|=|[X_k,V_k]| = |c_{2^k}-c_{3\cdot 2^k}| \approx b_k^2. \end{aligned}$$

These two statements imply $|(U_k,X_k)| \sim |[0,c_{2^k}]|\sim b_k$ and therefore (73). $\quad \square$

It follows from (74) that when $u_k\in R_k$ arbitrarily then $u_k\sim b_k$ as $k\rightarrow \infty$ and therefore we will be able to use $R_k$ instead of the intervals $X_k$ and $V_k$. Equation (72) will require us to choose much smaller intervals inside $W_k$.

### Lemma 15

Let $W_k^-$ and $W_k^+$ in $W_k$ which are mapped by $f_r \circ f_l$ onto $R_{k+2}$ resp. $L_{k+2}$, where we take $W_k^-$ is to the left of $W_k^+$. Then

75 $$\begin{aligned} \dfrac{|W_k^-|}{|W_k|}, \dfrac{|W_k^+|}{|W_k|} \rightarrow 0. \end{aligned}$$

Note that

76 $$\begin{aligned} \Lambda \cap [a_k,b_k] \subset W_k^- \cup W_k^+ \cup U_k \cup X_k \cup V_k. \end{aligned}$$

### Proof

Since (73) also holds for $k+2$ replaced by *k*, there exists four intervals in $U_k$ (with two in $L_{k+2}$ and two in $R_{k+2}$) so that the gap between $L_{k+2}$ and $R_{k+2}$ is huge compared to the size of these two intervals. Now consider the orientation reversing map $f_r \circ f_l:W_k\rightarrow U_k$. Since this map has bounded distortion (75) holds. $\quad \square$

Note that for each $x\in \Lambda \cap [a_k,b_k]$ either $x\in [a_{k+2},b_{k+2}]$ or *x* is contained in one of the sets $X_k$, $V_k$, $W_k^+$ or $W_k^-$. Moreover, as we have shown, if $u_k,v_k\in Q_k$ and $u_k\rightarrow 0$ where $Q_k$ is either $R_k=[X_k,V_k]$, $W_k^+$ or $W_k^-$ then $u_k \sim v_k$.

It remains to obtain asymptotic expressions for at least one point in each these intervals. Let us start with $W_k^+$. This interval contains a point $z_k$ so that $f_r\circ f_l(z_k)=0$. It follows that

$$\begin{aligned} |c_{2^{k+1}}-0|=|f_r(f_l(0))-f_r(f_l(z_k))| \sim \beta |f_l(0)-f_l(z_k)| \sim \beta |z_k|s_k. \end{aligned}$$

Since $s_k\sim \dfrac{b_k^{1-\beta }}{K_0}$ and $c_{2^{k+1}}\sim -\left[ \dfrac{K_0^\beta }{\beta ^{\beta +1}} \right] ^{1/(\beta -1)} b_k^{\beta +1}$ it follows that

77 $$\begin{aligned} z_k\sim - \dfrac{1}{\beta } \left[ \dfrac{K_0^\beta }{\beta ^{\beta +1}} \right] ^{1/(\beta -1)} b_k^{\beta +1} \dfrac{K_0}{b_k^{1-\beta }} = - \left[ \dfrac{K_0^{2\beta -1}}{\beta ^{2\beta }} \right] ^{1/(\beta -1)} b_k^{2\beta } . \end{aligned}$$

Similarly, $c_{2^{k+1}} \in W_k^-$ and according to (54)

78 $$\begin{aligned} c_{2^{k+1}}\sim -\left[ \dfrac{K_0^\beta }{\beta ^{\beta +1}} \right] ^{1/(\beta -1)} b_k^{\beta +1} . \end{aligned}$$

Finally, $$c_{3\cdot 2^k}, c_{2^k}\in R_k$$, by (43)

79 $$\begin{aligned} c_{3\cdot 2^k}\sim c_{2^k} \sim b_k . \end{aligned}$$

Let us now take the homeomorphism *h* between $\Lambda$ and $\tilde{\Lambda }$ defined so that $h(f^n(0))={{\tilde{f}}}^n(0)$ and show that *h* is differentiable at 0, provided that $\beta ={{\tilde{\beta }}}$, $\Theta ={{\tilde{\Theta }}}$ and $K_0={{\tilde{K}}}_0$. Because of these assumptions, Eq. (61) gives that for $k\rightarrow \infty$ even,

80 $$\begin{aligned} \dfrac{{{\tilde{b}}}_k}{b_k} \rightarrow \rho := \left[ \dfrac{K_0}{\tilde{K}_0}\right] ^{\frac{1}{\beta -1}} =1. \end{aligned}$$

Let $u_k\in \Lambda$ and take ${{\tilde{u}}}_k=h(u_k)$. By renumbering if necessary we may assume that $u_k\in W_k^-\cup W_k^+\cup X_k\cup V_k$. From (78) follows that for $u_k\in W_k^-$, $\tilde{u}_k\in {{\tilde{W}}}_k^-$,

$$\begin{aligned} {{\tilde{u}}}_k/u_k \rightarrow [{{\tilde{K}}}_0/K_0 ] ^{(2\beta -1)/(\beta -1)} (\tilde{b}_k/b_k)^{2\beta } \sim \rho ^{1-2\beta } \rho ^{2\beta }=\rho . \end{aligned}$$

From (77), $$u_k\in W_k^+$$, $${{\tilde{u}}}_k\in \tilde{W}_k^+$$,

$$\begin{aligned} {{\tilde{u}}}_k/u_k \rightarrow [{{\tilde{K}}}_0/K_0]^{\beta /(\beta -1)} ({{\tilde{b}}}_k/b_k)^{\beta +1} \sim \rho ^{-\beta } \rho ^{1+\beta }=\rho . \end{aligned}$$

Finally from (79) we have ${{\tilde{u}}}_k/u_k\rightarrow \rho$ for $u_k\in X_k\cup V_k$ and ${{\tilde{u}}}_k\in {{\tilde{X}}}_k\cup {{\tilde{V}}}_k$. It follows that $h:\Lambda \rightarrow {{\tilde{\Lambda }}}$ is differentiable at 0.

**The invariants** (68) **are sufficient for the conjugacy** $h:\Lambda \rightarrow {{\tilde{\Lambda }}}$**to be differentiable along** $\Lambda$, **where** $\Lambda =\overline{\cup _{n\ge 0} f^n(0)}$. Let $\Delta _{k,0}=[a_k,b_k]$, $\Delta _{k,i}=f^i(\Delta _k^0)$, $i=1,\dots ,2^k-1$ and $\Delta _k=\cup _{0\le i\le 2^k-1} \Delta _{k,i}$. Note that $\Lambda =\cap _k \Delta _k$. Moreover, let ${{\tilde{\Delta }}}_{k,i},{{\tilde{\Delta }}}_k$ be the corresponding the sets for ${{\tilde{f}}}$. As in [47, Section VI.9], define $\Omega =\{0,1\}^\mathbb N$ and a continuous map $\phi :\Omega \rightarrow \Lambda$ defined by associating to $\omega \in \Omega =\{0,1\}^\mathbb N$ the point $\cap _k \Delta ^{j(k,\omega )}$ where $j(k,\omega )=\sum _{i=0}^{k-1} \omega (i) 2^j$. Denote the interval $\Delta _{k,j(k,\omega )}$ by $[\omega (0),\dots ,\omega (k-1)]_k$ and let $[\omega (0),\dots ,\omega (k-1)]_{k,\sim }$ be the corresponding interval for ${{\tilde{f}}}$. Because *f* has the period doubling combinatorics,

$$\begin{aligned}{}[\omega (0),\dots ,\omega (k-1)]_k\subset [\omega (0),\dots ,\omega (k-2)]_{k-1}. \end{aligned}$$

Let $\Omega ^*$ be the dual Cantor set consisting of all left infinite words

$$\begin{aligned} \left\{ \omega =\left( \dots ,\omega (k),\dots ,\omega (1),\omega (0)\right) , \omega (i)\in \{0,1\}\right\} \end{aligned}$$

with the product topology. From the scaling law (61) we obtain that

$$\begin{aligned} \dfrac{[0,\dots ,0,0,0]_{k+2}}{[0,\dots ,0,0]_{k}}= (1+\epsilon _k) \exp (2^k(\Theta -4\Theta )). \end{aligned}$$

From the calculation in (59)–(61) it follows that $\prod _{n\ge k}(1+\epsilon _n)$ goes to one as $k\rightarrow \infty$. (In fact, one can show that $\epsilon _n$ tends exponentially fast to zero.) From the above consideration we also have that for $j_1,j_2\in \{0,1\}$

$$\begin{aligned} \dfrac{[0,\dots ,0,j_1,j_2]_{k+2}}{[0,\dots ,0,0]_{k}} = (1+\epsilon _k) \kappa (\beta ,j_1,j_2) \exp (-2^k\Psi (\Theta ,\beta ,j_1,j_2)) \end{aligned}$$

where $\kappa (\beta , j_1,j_2)>0$ and $\Psi (\Theta ,\beta ,j_1,j_2)$ are constants which can be computed explicitly as above (and which only depend on $\beta ,\Theta ,j_1,j_2$). Using the fact that the Koebe space of the semi-extension of the first entry map from $\Delta _k^i$ into $\Delta _{k,2^k}\subset \Delta _{k,0}$ tends exponentially fast to infinity, and therefore the non-linearity of the first entry map tends exponentially fast to zero, we obtain

$$\begin{aligned} \dfrac{[\omega (k+1),\dots ,\omega (2),j_1,j_2]_{k+2}}{[\omega (k+1),\dots ,\omega (2)]_{k}} = (1+\epsilon _k) \kappa (\beta ,j_1,j_2) \exp (-2^k\Psi (\Theta ,\beta ,j_1,j_2)). \end{aligned}$$

Hence, as in [47, Proof of Theorems VI.9.3 and VI.9.1], using the property that $\prod _{n\ge k}(1+\epsilon _n)$ converges to 1 as $k\rightarrow \infty$ and assuming that (68) holds we obtain that for each sequence $\omega \in \Omega ^*$

$$\begin{aligned} \dfrac{[\omega (k-1),\dots ,\omega (0)]_{k,\sim }}{[\omega (k-1),\dots ,\omega (0)]_{k}} \end{aligned}$$

converges and the value of the limit depends continuously on $\omega \in \Omega ^*$. From this it follows that the conjugacy is differentiable along $\Lambda$.

## The Hausdorff Dimension of the Attracting Cantor Set is Zero

Recall that for every $k>0$ and $i=0,\ldots , 2^k-1$ we have defined $\Delta _{k,i}:= f^i([a_k,b_k])$.

Let us make a few observations on locations of certain intervals $\Delta$ inside their parents. In what follows *k* is assumed to be even. First, observe that the both intervals $\Delta _{k+2, 2^k}$ and $\Delta _{k+2, 3\cdot 2^k}$ belong to $[c_{3\cdot 2^k},c_{2^k}]$. Secondly, $\Delta _{k+2, 2\cdot 2^k} \subset [c_{2\cdot 2^k}, c_{4\cdot 2^k}]$. Also note that all 4 mentioned intervals belong to $\Delta _{k,0}$.

Using formulas (41), (42) and (51) we see that $|\Delta | < C|\Delta _{k,2^k}|^2$ for $\Delta =\Delta _{k+2, 2^k}$, $\Delta _{k+2, 2\cdot 2^k}$, $\Delta _{k+2, 3\cdot 2^k}$, $\Delta _{k+2, 4\cdot 2^k}$, where *C* is some universal constant.

Fix some integer $1\le i \le 2^k-1$. The distortion of the map $f^{2^k-i}: \Delta _{k,i} \rightarrow \Delta _{k,0}$ is asymptotically small due to Theorem 3 and Lemma 1 (*k* is still assumed even). We know that $f^{2^k-i}(\Delta _{k,i}) = [a_k, c_{2^k}]$ and this interval is very close to $\Delta _{k,0}:=[a_k,b_k]$ due to formula (6). Hence, if $\Delta \subset \Delta _{k,i}$ is one of four intervals of the form $\Delta _{k+2, m}$, then $|\Delta | < C|\Delta _{k, 0}||\Delta _{k,i}|$, where *C* is another universal constant. This estimate implies that for any $\gamma >0$ there exists $k_0$ (depending on *f*) such that if $k>k_0$ and *k* is even, $|\Delta |^\gamma < \frac{1}{4} |\Delta _{k, i}|^\gamma$. Therefore,

$$\begin{aligned} \sum _{i=0}^{4\cdot 2^k-1} |\Delta _{k+2, i}|^\gamma < \sum _{i=0}^{2^k-1} |\Delta _{k, i}|^\gamma . \end{aligned}$$

Thus we have shown that the Hausdorff dimension of $\Lambda$ is zero.

## Absence of any Koebe Space for General First Entry Maps

Define $R_k$ to be the first return map to $[a_k,b_k]$.

### Theorem 13

(Theorem 9 - Absence of Koebe space) For each $\tau >0$ there exists *x* and *k* so that the maximal semi-extension of the first entry map from *x* into $[a_k,b_k]$ does **not** contain a $\tau$-scaled neighbourhood of $[a_k,b_k]$.

### Proof

Assume that $x\in I$ and *n* is so that $y=f^n(x)$ is a first entry to $[a_{2i-1},b_{2i-1}]$ and that in fact $y\in [b_{2i},b_{2i-1}]$. Moreover, assume that $y'=R_{2i-1}(y)\in [a_{2i},b_{2i}]$. Write $y'=f^m(x)$ so $y'$ is a first entry of *x* into $[a_{2i},b_{2i}]$ under $f^m$. Since $f^m=R_{2i-1}\circ f^n$, the maximal diffeomorphic extension (or even semi-extension) of $f^m$ is at most that of $R_{2i-1}$. The diffeomorphic range of the latter map is $[c_{2^{2i-1}},B_{2i-1}]$. By Theorem 4 we have $c_{2^{2i-1}}\approx -b_{2i-1}^{\beta +1}$.

The length of $[a_{2i},b_{2i}]$ is $\sim b_{2i}\approx b_{2i+1} \approx b_{2i-1}^2$, and since $\beta >1$, therefore the space $[c_{2^{2i-1}},a_{2i}]$ is minute compared to the size of the interval $[a_{2i},b_{2i}]$ when *i* large. It follows that when *i* is large, there exists no $\tau >1$ so that the range of the extension $[c_{2^{2i-1}},B_{2i-1}]$ contains a $\tau$-scaled neighbourhood of $[a_{2i},b_{2i}]$. In fact, the range of the extension is also not a $\tau$-scaled neighbourhood of $[a_{2i+1},b_{2i+1}]$ for the same reason. $\quad \square$

## Absence of Wandering Intervals

### Lemma 16

(The orbit of a potential wandering interval) If *f* has a wandering interval *W*, then

1. $W_k:=f^k(W)$ accumulates onto 0, so for some sequence of $k_j$’s tending to infinity $W_{k_j}\rightarrow 0$;
2. there exists $i_0$ so that if $W_k\subset {[}a_{2i_0-1},b_{2i_0-1}]$ for some *k* then $W_k \subset \bigcup _{i\ge i_0} [b_{2i},b_{2i-1}]$;
3. if $W_k\subset [b_{2i},b_{2i-1}]$ then $W_k\subset [b_{2i},\eta _ib_{2i-1}]$ where $\eta _i \rightarrow 0$ as $i\rightarrow \infty$.

### Proof

The sequence of intervals $W_i:=f^i(W)$ must accumulate to 0 for some subsequence $i_j\rightarrow \infty$. Indeed, otherwise there exists a small neighbourhood $U_0$ of 0 and $n_0\ge 0$ so that $f^n(W)\cap U_0= \emptyset$ for all $n\ge n_0$. But a theorem of Mañé, see [46][Theorem III.5.1] implies that there exists $K>0, \lambda >1$ so that $|Df^n(x)|\ge K\lambda ^n$ for all $x\in [a_0,b_0]$ so that $f^i(x)\notin U_0$ for $i=0,\dots ,n-1$. Hence the length of the disjoint intervals $f^n(W)$ is growing exponentially with *n*, which of course is a contradiction. It follows that $W_i\not \ni 0$ for all $i\ge 0$. So for any *k* there exists a *minimal* $n(k)\ge 0$ so that $W_{n(k)}\subset I_k=[a_k,b_k]$ where $n(k)\rightarrow \infty$ as $k\rightarrow \infty$. Since all iterates of *W* are disjoint, $W_i\cap \{a_k,b_k\}=\emptyset$ for all $i\ge 0, k\ge 0$.

By minimality of *n*(*k*), $W_i\cap [a_k,b_k]=\emptyset$ for all $i<n(k)$. Hence if we take $T_k\supset W$ to be the maximal interval so that $f^{n(k)}|T_k$ is a diffeomorphism then by Lemma 3 there exists $\tau >1$ so that $f^{n(k)}(T_k)$ contains $[\tau a_k,\tau b_k]$.

(1) Let us first show that $W_{n(k)}$ lies to the right of 0 for all *k* large. Indeed, assume by contradiction that there exists infinitely many *k*’s so that $W_{n(k)}\subset [a_k,0]$. For each such *k*, $f^{n(k)}(T_k)\supset [\tau a_k,\tau b_k]$ is a scaled-neighbourhood of $W_{n(k)}$. By Koebe it follows that $T_k$ also contains a $\tau '$-scaled neighbourhood of *W* where $\tau '>0$ is the same for infinitely many *k*’s. This shows that there exists an interval $W'\supset W$ which strictly contains *W* on which all iterates of *f* are diffeomorphic, contradicting the maximality of *W*.

**Fig. 8:**
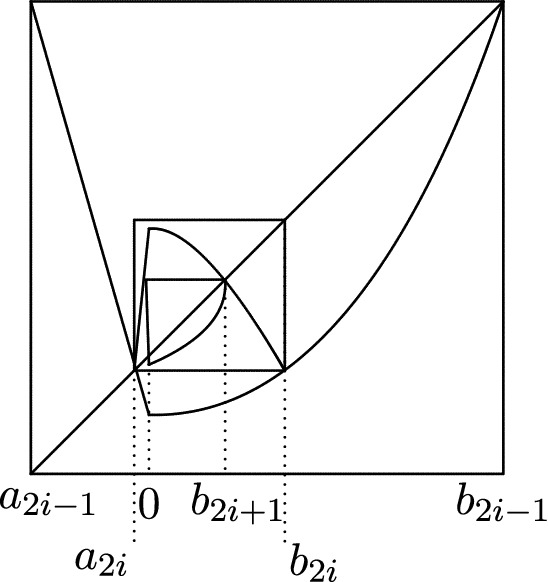
The return maps $R_j$ to $[a_j,b_j]$ for $j=2i-1,2i,2i+1$

(2) Let us now show that there exists $k_0$ so that if $k\ge k_0$ is even then $W_{n(k)}$ cannot be contained in $[b_{k+1},b_k]$. Indeed, when *k* is even then by Theorem 4, $[\tau a_k,\tau b_k]$ is a scaled neighbourhood of $[b_{k+1},b_k]$ and so as in the previous case we obtain a contradiction.

From (1) and (2) it follows that for all *k* large, $W_{n(k)}$ is contained in $\bigcup _i [b_{2i},b_{2i-1}]$. Similarly to (2), we have that if $W_{n(k)}$ is contained in $[b_{2i},b_{2i-1}]$ then in fact it is contained in $[b_{2i},\eta b_{2i-1}]$ where $\eta \in (0,1)$ is small when *i* is large. Here we use that $W_{n(k)}$ must be contained in a fundamental domain of the fixed point $b_{2i-1}$ of $R_{2i-1}$. $\quad \square$

As above let $n(k)\ge 0$ be minimal so that $W_{n(k)}\subset I_k=[a_k,b_k]$. From the previous lemma it follows that $W_{n(k)}$ is contained in $[b_{2i},b_{2i-1}]$ for some $2i-1\ge k$ and therefore $n(2i-1)=n(k)$. The first return map $R_{2i-1}$ to $[a_{2i-1},b_{2i-1}]$ is drawn in Fig. 8 on page 47 and satisfies $R_{2i-1}(x)<x$ for $x\in [0,b_{2i-1}]$. It follows that there exists $m_k\ge 1$ so that

81 $$\begin{aligned} R_{2i-1}^j(W_{n(k)}) \subset [b_{2i},b_{2i-1}] \text{ for } \text{ all } 0\le j < m_k \end{aligned}$$

and then for some $i'>i$,

82 $$\begin{aligned} R_{2i-1}^{m_k}(W_{n(k)})\subset [b_{2i'},b_{2i'-1}]. \end{aligned}$$

In other words, the next first entry into $$[a_{2i},b_{2i}]$$ is in fact into $$ [b_{2i'},b_{2i'-1}]$$ and in particular $$n(2i-1)<n(2i)=\dots =n(2i'-1)$$.

### Lemma 17

*f* does not have wandering intervals.

### Proof

Let us write $R_{2i-1}=\phi _{2i-1}(x^\beta )$ on $[0,b_{2i-1}]$ where $\phi _{2i-1}$ is an orientation preserving diffeomorphism. For convenience we will write $\phi$ rather than $\phi _{2i-1}$. Let us first obtain an estimate for $\phi$. It follows from Lemma 12 and part (3) of Lemma 16$|\phi '(x)/\phi '({{\hat{x}}})-1|\le \epsilon$ for all $x,{{\hat{x}}}\in [b_{2i}^\beta ,\eta b_{2i-1}^\beta ]$ where $\epsilon >0$ is small when $\eta$ is small and *i* is large. It follows that there exists $\gamma >0$ so that

83 $$\begin{aligned} - \gamma \epsilon \le \phi '(x)-\gamma \le \gamma \epsilon . \end{aligned}$$

Since $\phi (0) =c_{2^{2i-1}}<0$ it follows that

84 $$\begin{aligned} \phi (0) + (1-\epsilon )\gamma x \le \phi (x)\le \phi (0)+ (1+\epsilon )\gamma x \le (1+\epsilon )\gamma x. \end{aligned}$$

Note that $|c_{2^{2i-1}}|\approx | b_{2i-1}^{\beta +1}|<<|b_{2i-1}|$ and therefore $R_{2i-1}(b_{2i-1})=b_{2i-1}$ implies that $\gamma \approx b_{2i-1}^{1-\beta }$.

From (60) we have $\log (1/b_{2i-1}) \approx 2^i$, $\log (1/b_{2i}) \approx 2^{i+1}$, and therefore $\log (\log (1/b_{2i-1})) \approx i \log 2 + O(1)$, $\log (\log (1/b_{2i})) \approx (i+1) \log 2 + O(1)$ and so the length of the intervals $[b_{2i},b_{2i-1}]$ is bounded in double logarithmic coordinates.

Let us show that $R_{2i-1}$ is expanding in double logarithmic coordinates. So define $l_2(x)=\log (\log (1/x))$ where we assume $x\in [b_{2i},\eta b_{2i-1}]$. Then

$$\begin{aligned} Dl_2(x)=\dfrac{-1}{x\log (1/x)} \text{ and } x=l_2^{-1}(y)=e^{-e^y}. \end{aligned}$$

Moreover,

$$\begin{aligned} D(l_2 \circ R_{2i-1}\circ l_2^{-1})(y)= D(l_2 \circ \phi \circ f \circ l_2^{-1} )(y)= \dfrac{\phi '(e^{-\beta e^y}) (\beta e^y) e^{-\beta e^y}}{\phi (e^{-\beta e^y})\log (1/\phi (e^{-\beta e^y}))}. \end{aligned}$$

Since $x=l_2^{-1}(y)=e^{-e^y}$, $\log x=-e^y$ and $\log (1/x^\beta )=\beta e^y$ this is equal to

$$\begin{aligned} \dfrac{\phi '(x^\beta ) x^\beta \log (1/x^\beta )}{\phi (x^\beta ) \log (1/\phi (x^\beta )) } \ge (1-\epsilon ) \gamma \dfrac{x^\beta \log (1/x^\beta )}{\phi (x^\beta ) \log (1/\phi (x^\beta )) } \end{aligned}$$

where in the inequality we used (83). Since $t\mapsto t \log ( 1/t)$ is increasing for $t>0$ small and because of (84) the latter expression is bounded below by

$$\begin{aligned} \ge (1-\epsilon )\gamma \dfrac{x^\beta \log (1/x^\beta )}{(1+\epsilon )\gamma x^\beta \log (1/((1+\epsilon )\gamma x^\beta ))} = \dfrac{ (1-\epsilon )}{(1+\epsilon )} \dfrac{\log (1/x^\beta )}{\log (1/((1+\epsilon )\gamma x^\beta ))} . \end{aligned}$$

Since $\gamma \approx b_{2i-1}^{1-\beta }$, there exists $C_0>0$ so that this is bounded below by

$$\begin{aligned} \ge \dfrac{1-\epsilon }{1+\epsilon } \,\, \dfrac{\log (1/x^\beta )}{\log (1/x^\beta ) + (1-\beta )\log (1/b_{2i-1})+ \log (C_0)}. \end{aligned}$$

Since the latter expression is increasing in *x* for $x\in [0,b_{2i-1}]$ and since $x\in [b_{2i},b_{2i-1}]$ this is bounded from below by

$$\begin{aligned} \dfrac{1-\epsilon }{1+\epsilon } \,\, \dfrac{\beta \log (1/b_{2i})}{\beta \log (1/b_{2i}) + (1-\beta )\log (1/b_{2i-1}) + \log (C_0)} . \end{aligned}$$

Since $b_{2i}\approx b_{2i-1}^2$ this is bounded from below by

$$\begin{aligned} \dfrac{1-\epsilon }{1+\epsilon } \,\, \dfrac{2\beta \log (1/b_{2i-1}) + \log (C_0'')}{2\beta \log (1/b_{2i-1}) + (1-\beta )\log (1/b_{2i-1}) + \log (C_0')} \ge \dfrac{2\beta }{1+\beta }- o(\epsilon )>1 \end{aligned}$$

provided *i* is large and $\epsilon >0$ is small. It follows that in double-logarithmic coordinates $R_{2i-1}$ is expanding on $[b_{2i},\eta b_{2i-1}]$.

It follows that if *W* is a wandering interval above, then in double-logarithmic coordinates the iterates described in (81) and (82) increase each step in length by a factor $(\beta +1)/2$. So their length tends to infinity. But this violates that all iterates are contained in $\cup _{i\ge i_0} [b_{2i},b_{2i-1}]$ because, as we saw, in double-logarathmic coordinates the length of the intervals $[b_{2i},b_{2i-1}]$ is uniformly bounded from above. $\quad \square$
